# Ndk, a novel host-responsive regulator, negatively regulates bacterial virulence through quorum sensing in *Pseudomonas aeruginosa*

**DOI:** 10.1038/srep28684

**Published:** 2016-06-27

**Authors:** Hua Yu, Junzhi Xiong, Rong Zhang, Xiaomei Hu, Jing Qiu, Di Zhang, Xiaohui Xu, Rong Xin, Xiaomei He, Wei Xie, Halei Sheng, Qian Chen, Le Zhang, Xiancai Rao, Kebin Zhang

**Affiliations:** 1 Department of Microbiology, College of Basic Medical Sciences, Third Military Medical University, Chongqing, China; 2 Central Laboratory, Xinqiao Hospital, Third Military Medical University, Chongqing, China; 3 Department of Pharmacy, Xinqiao Hospital, Third Military Medical University, Chongqing, China

## Abstract

Pathogenic bacteria could adjust gene expression to enable their survival in the distinct host environment. However, the mechanism by which bacteria adapt to the host environment is not well described. In this study, we demonstrated that nucleoside diphosphate kinase (Ndk) of *Pseudomonas aeruginosa* is critical for adjusting the bacterial virulence determinants during infection. *Ndk* expression was down-regulated in the pulmonary alveoli of a mouse model of acute pneumonia. Knockout of *ndk* up-regulated transcription factor ExsA-mediated T3S regulon expression and decreased exoproduct-related gene expression through the inhibition of the quorum sensing hierarchy. Moreover, *in vitro* and *in vivo* studies demonstrated that the *ndk* mutant exhibits enhanced cytotoxicity and host pathogenicity by increasing T3SS proteins. Taken together, our data reveal that *ndk* is a critical novel host-responsive gene required for coordinating *P. aeruginosa* virulence upon acute infection.

*Pseudomonas aeruginosa* is a versatile Gram-negative opportunistic pathogen responsible for causing acute and chronic infections. It has the largest bacterial genome (more than 6.2 million base pairs) in which 8.4% of the genome encodes transcriptional regulators and environmental sensors, which renders the bacteria highly adaptive to overcome hostile conditions^1^. The capacity of *P. aeruginosa* to perceive the host environmental stresses and to make adaptive responses enables the bacteria to survive and establish infection^2^. Nevertheless, the investigations of how bacteria sense host environmental stimuli and make adjustments are limited.

Acute lung infection caused by *P. aeruginosa* is a great threat for human health and is associated with poor clinical outcome. The secreted virulence products, such as elastase (LasA, LasB), alkaline proteinase, exotoxin A, and phospholipase C, play important roles in *P. aeruginosa*-mediated acute lung epithelial injury^3^. The type III secretion system (T3SS) is a vital virulence determinant in promoting acute lung infection via *P. aeruginosa*^4^.The T3SS is a complex protein secretion and translocation machinery composed of five protein components, including secretion apparatus (termed the injectisome), translocation apparatus, regulatory proteins, effector proteins and chaperones^5^. The underlying mechanism of T3SS-mediated virulence relies on an arsenal of toxic effector proteins that are directly injected into the cytoplasms of eukaryotic cells by the T3SS syringe-like injectisome^5^,^6^. Four effectors, termed ExoS, ExoT, ExoY and ExoU, have been identified in *P. aeruginosa*^6^. These effectors are involved in a variety of bacterial virulence associated activities, such as the induction of host cell death, the inhibition of cell proliferation, the preclusion of cell phagocytosis, and the disturbance of the host innate immune response^5^,^6^. In addition, T3SS exhibits an exotoxin S/T/Y-independent pathogenic role during acute lung infection^7^. The T3SS translocation apparatus (constituted by translocators PopB, PopD, and PcrV), which aids in delivering the effector proteins across the host cell plasma membrane, is sufficient to cause eukaryotic cell death by directly increasing the membrane permeability response^5^.

It has been reported that *P. aeruginosa* T3SS regulation is involved in a series of complicated mechanisms, such as the direct effect on T3SS-encoding gene expression and type III secretion activity^8^. Transcription factors, such as ExsA^9^ and PsrA^10^, are direct activators that promote the transcription of T3SS-encoding genes. Other proteins, such as RNA-binding protein Crc^11^ and DeaD (PA2840) (an *Escherichia coli* RNA-helicase homologue)^12^ fine tune *P. aeruginosa* T3SS gene expression by regulating ExsA. Apart from the direct regulation of T3SS gene transcription, T3SS activation is coupled with type III secretion activity in different conditions, such as host cell contact and low Ca^2+^ signaling, that activate the ExsCEDA regulatory cascade by promoting ExsE secretion^8^.

Quorum sensing (QS) is a cell-density-dependent mechanism of communication among microorganisms. In *P. aeruginosa*, three QS regulatory circuits have been reported, including two *N*-acylhomoserine lactone (AHL) regulatory circuits (*las* and *rhl* systems) and a quinolone signaling circuit (*pqs* system)^13^. In the *las, rhl* and *pqs* systems, the LasI and RhlI synthetases and *pqsABCD* gene products catalyze the synthesis of signal molecules *N*-(3-oxododecanoyl)-l-homoserine lactone (3-oxo-C12-HSL), *N*-butanoyl-l-homoserine lactone (C4-HSL) and 2-heptyl-3-hydroxy-4-quinolone (PQS), respectively^13^. When 3-oxo-C12-HSL, C4-HSL and PQS interact with their cognate transcriptional regulators LasR, RhlR and PqsR, respectively, the complex modulates the expression levels of its down-stream genes involved in T3SS protein synthesis and the production of elastase, pyocyanin, LasA protease, alkaline protease, exotoxin A and siderophores^13^,^14^,^15^. Multiple factors are involved in the regulation of the *P. aeruginosa* QS system, such as the regulator proteins QteE^16^, VqsM^17^ and QsrO (QS-repressing ORF2226)^18^, and the quorum quenching acylases PvdQ and PA0305^19^,^20^.

Nucleoside diphosphate kinase (Ndk), a vital NTP-generating kinase in the maintenance of intracellular NTP pools via its terminal phosphotransfer activity from NTPs to NDPs, shares highly homology among microorganisms, humans, animals, and plants^21^. Ndk is secreted outside of the bacteria; thus, its direct host pathogenicity has been extensively studied. In *Mycobacterium tuberculosis*, secreted Ndk promotes bacterial survival within host macrophages by arresting phagosome maturation through GTPase activating protein (GAP) activity or by disturbing host gene expression via its DNAse activity^22^. Ndk can be transported into host cells by the T3SS apparatus in *P. aeruginosa*^23^, and it functions as a T3SS effector in inducing the host inflammatory response via stimulating the secretion of interleukin-1α (IL-1α), IL-1β, and IL-8^24^,^25^. In spite of previous reports that showed that Ndk plays critical roles in host pathogenicity and the regulation of bacterial virulence-associated traits, such as DNA/RNA synthesis, cell division and alginate synthesis^21^,^26^, the available data showing that Ndk contributes to the regulation of bacterial virulence, particularly the virulence of the notorious *P. aeruginosa*, are still limited.

In this study, it was found that *ndk* is a novel host-responsive regulator that is down-regulated in the pulmonary alveoli of a mouse model of acute pneumonia. An *ndk-*deleted mutant promoted *P. aeruginosa* cytotoxicity and host pathogenicity by activating T3SS. Further analysis revealed that Ndk inhibits T3SS through the QS-ExsA circuit. Therefore, a better understanding of Ndk and its regulatory role in bacterial virulence will maximize opportunities for the development of strategies to treat *P. aeruginosa* infection.

## Results

### Transcription of T3SS components was activated whereas *ndk* was inhibited in *P. aeruginosa* in a mouse model of acute pneumonia

**Figure 1:**
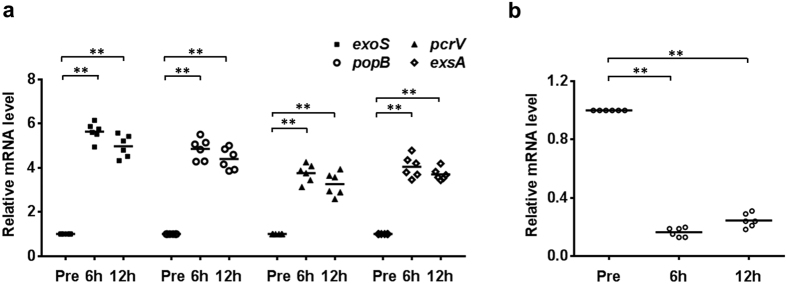
Transcription of T3SS components was activated whereas *ndk* was inhibited in *P. aeruginosa* in a mouse model of acute pneumonia. Mice were intranasally challenged with 2 × 10^8^ CFU of PAO1. At 6 and 12 h post-infection, the BALFs were collected from the infected mice and the expression levels of T3SS genes (**a**) and *ndk* (**b**) in BALFs were evaluated by real-time PCR. The 50S ribosomal protein-coding gene *rplU* was used as an internal control. Black bars represent medians for the group of mice. In each time point, six mice were used. *Represents *P* < 0.05 compared to bacteria *in vitro* (Pre).

Upon acute infection, the activation of type III secretion machinery is coupled with increased T3SS gene transcription^8^, which compelled us to investigate the expression levels of T3SS-encoding genes in *P. aeruginosa* during acute pneumonia. Initially, mice were intranasally challenged with PAO1. At 6 and 12 h post-infection, the bronchoalveolar lavage fluid (BALF) was collected, and the expression levels of T3SS genes were evaluated by real-time PCR. The genes encoding the T3SS effector protein ExoS, the translocation apparatus (PcrV and PopB) and the transcription factor ExsA were drastically up-regulated in *P. aeruginosa* during infection compared to those of *P. aeruginosa* cultured *in vitro* (Fig. 1a).

In addition, to test whether *ndk* might function as a responsive gene during *P. aeruginosa* acute infection, *ndk* expression in BALF was investigated. As shown in Fig. 1b, *ndk* expression was significantly down-regulated during *P. aeruginosa* infection. These results hint that a connection between *ndk* and T3SS might exist during *P. aeruginosa* acute infection.

Given that host cell contact is a crucial factor to activate T3SS gene expression upon *P. aeruginosa* infection^8^, we further examined whether the inhibition of *ndk* expression during pulmonary infection of *P. aeruginosa* is in a cell contact-dependent manner. To verify this speculation, the PAO1 strain was utilized to infect paraformaldehyde fixed or non-fixed human alveolar basal epithelial adenocarcinomic A549 cells and murine RAW264.7 macrophage cell lines. Unexpectedly, the *ndk* expression level did not show apparent change in the non-fixed and fixed cells compared to PAO1 cultured in DMEM (Supplementary Fig. 1), indicating that the inhibition of *ndk* expression during pulmonary infection of *P. aeruginosa* is not dependent on the host cell contact.

### The *ndk* null-mutant of *P. aeruginosa* exacerbates host pathogenicity *in vivo* by T3SS activation

**Figure 2:**
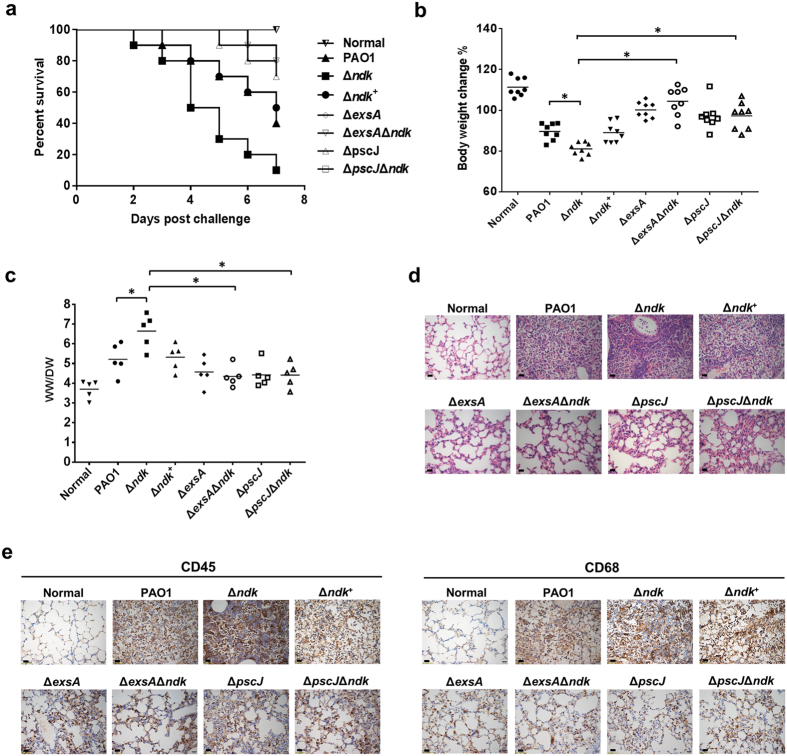
The *ndk* null-mutant of *P. aeruginosa* exacerbates host pathogenicity *in vivo* by T3SS activation. Mice were intranasally challenged with the indicated strains (0.5 × 10^8^ CFU per mouse). The subsequent analyses were evaluated at 7 days post-infection. (**a**) Mouse survival rate. Ten mice were included in each group. Survival curves were estimated using the Kaplan-Meier analysis with a log-rank test. (**b**) Body weight change. Eight mice were included in each group. (**c**) Lung edema. Lung edema was evaluated by calculating the lung wet-to-dry weight ratio. Five mice were included in each group. *Represents *P* < 0.05. Black bars represent medians for the group of mice. (**d**) Photomicrographs of mouse lung. The lung tissues embedded in paraffin were cut in 5 μm thickness and were subjected to H&E staining. Scale bar: 20 μm. (**e**) IHC analysis of inflammatory cell infiltration. The leukocyte common antigen CD45 was used for the detection of leukocytes, and CD68 was used for the visualization of macrophage. Scale bar: 20 μm.

To address whether *ndk* expression is connected with T3SS in host pathogenicity, the mouse survival rate, body weight, lung edema and histopathology were evaluated in mice intranasally challenged with *P. aeruginosa* strain PAO1, a chromosomal *ndk* deletion mutant (Δ*ndk*), and an *ndk* complementary strain (Δ*ndk*^+^). As shown in Fig. 2a, Δ*ndk* caused 90% mortality, which was much higher than that caused by PAO1 and Δ*ndk*^+^ (60% and 50%, respectively) at 7 days post-infection. In addition, the mice infected with Δ*ndk* suffered a greater loss of body weight (Fig. 2b) and displayed increased lung edema (Fig. 2c) compared to the PAO1- and Δ*ndk*^+^-infected mice. Histopathology determined by hematoxylin and eosin (H&E) staining showed that lungs from Δ*ndk*-infected mice displayed more obvious inflammatory cell infiltration than the PAO1- and Δ*ndk*^+^-infected groups (Fig. 2d). Further analysis by immunohistochemical (IHC) staining showed that more leukocytes and macrophages infiltrated the lungs of Δ*ndk*-infected mice compared to the PAO1- and Δ*ndk*^+^-infected groups (Fig. 2e).

To further investigate whether the increased pathogenicity of Δ*ndk* is related to activated T3SS upon *P. aeruginosa* infection, the mice were intranasally challenged with the reduced T3SS expression strain Δ*exsA*, the T3SS effector deficient strain Δ*pscJ* (defective in type III secretion^27^), and the double knock-out strains Δ*exsA*Δ*ndk* and Δ*pscJ*Δ*ndk*. Compared to PAO1-infected mice, the Δ*exsA-* and Δ*pscJ*-infected mice showed significantly decreased mortality (Fig. 2a), body weight loss (Fig. 2b), lung edema (Fig. 2c), and lung inflammatory cell infiltration (Fig. 2d). Similar results were also observed in Δ*exsA*Δ*ndk-* and Δ*pscJ*Δ*ndk*-infected mice when compared to Δ*ndk*-infected mice (Fig. 2), suggesting that the inhibition of *ndk* expression might lead to increased host pathogenicity through T3SS activation.

### The *ndk* null-mutant enhances *P. aeruginosa* virulence in A549 cells through T3SS activation

**Figure 3:**
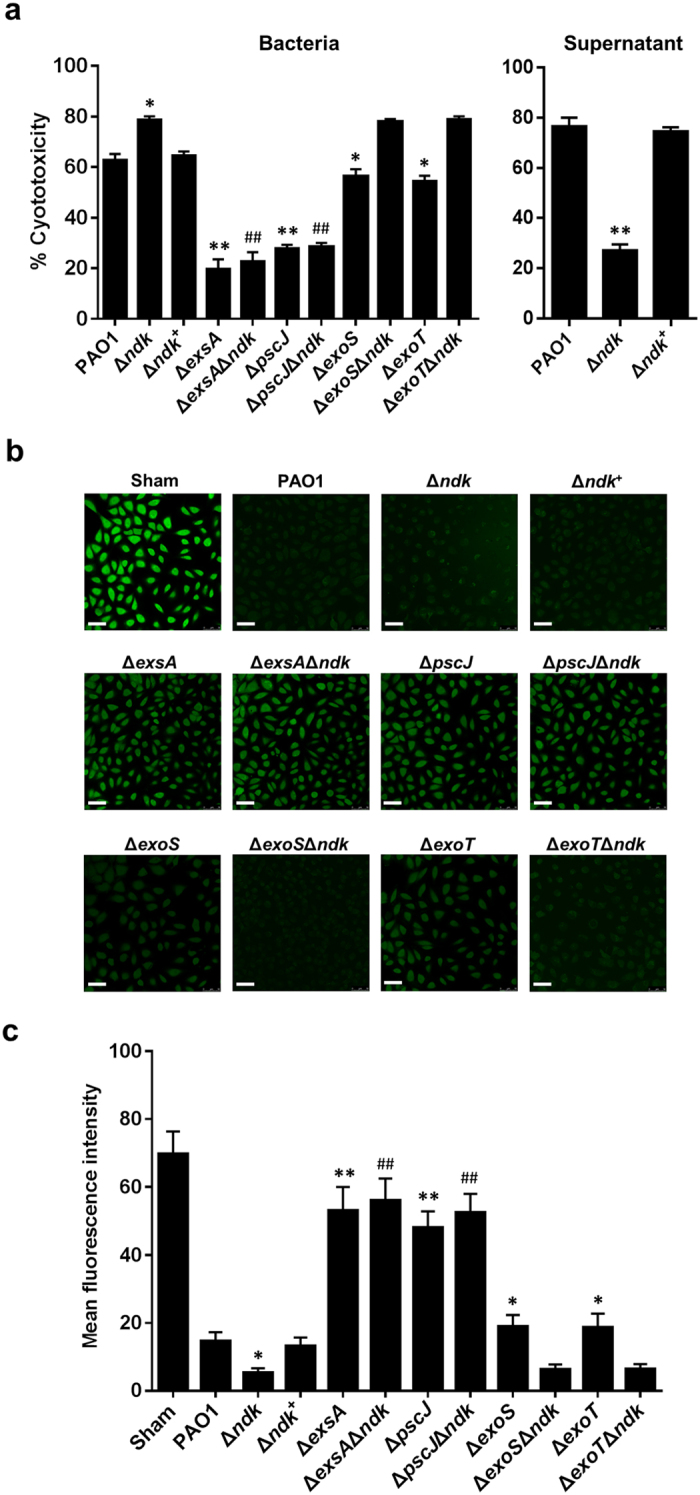
The *ndk* null-mutant enhances *P. aeruginosa* virulence in A549 cells through T3SS activation. Prior to cell infection, the indicated bacterial strains were cultured in LB broth for 12 h. The bacterial pellets and culture supernatants were collected after centrifugation. A549 cells were infected with the indicated strains (MOI = 50). In addition, A549 cells were treated with bacterial culture media 5-fold diluted in DMEM. At 2 h post-infection, the cell viability was detected. (**a**) CCK-8 assay. The percentage of cytotoxicity was calculated according to the following formula: cytotoxicity % = 1 − (OD_450 infected cells_/OD_450 sham-infected control_) × 100%. (**b**) Calcein-AM staining. Scale bar: 50 μm. Representative images from triplicate experiments are shown. (**c**) Fluorescence intensity per cell from (**b**) was analyzed by Image pro-Plus software (IPP, edition 6.0) (n_cell_ = 90). Data are expressed as the mean ± SD from three independent experiments. *Indicates *P* < 0.05 compared to PAO1 strain. ^#^Indicates *P* < 0.05 compared to Δ*ndk* strain.

Based on the *in vivo* study, the influence of *ndk* expression on *P. aeruginosa* virulence was further investigated *in vitro*. A549 cells were infected with PAO1, Δ*ndk*, and Δ*ndk*^+^, and cell viability was analyzed with a Cell Counting Kit 8 (CCK8) assay and calcein-AM staining. The CCK8 assay demonstrated that Δ*ndk* caused a much higher cytotoxicity than PAO1 and Δ*ndk*^+^ (Fig. 3a). Calcein-AM is a fluorescence stain for viable cells; the cell viability was estimated by calculating the green fluorescence intensity^28^. In accordance with the CCK8 assay, the result from calcein-AM staining also showed a similar trend (Fig. 3b,c). Given that *P. aeruginosa* secretes a variety of exoproducts that are toxic to cells, we examined whether the virulence products secreted by the bacteria were also involved in cytotoxicity during infection. To verify this speculation, the supernatants from PAO1, Δ*ndk*, and Δ*ndk*^+^ cultures were utilized to infect A549 cells. The cytotoxicity of the Δ*ndk* culture medium was significantly lower than those from PAO1 and Δ*ndk*^+^ media (Fig. 3a,b), indicating that the increased cytotoxicity of Δ*ndk* might be due to the changes of virulence-related determinants other than secretion products.

In view of the above observations, we sought to determine whether T3SS is involved in the cytotoxicity of Δ*ndk*. Therefore, T3SS gene knockout strains were applied to infect A549 cells. Compared to PAO1-infected cells, the cytotoxicity was much lower in Δ*exsA-* and Δ*pscJ-*infected cells (Fig. 3). Moreover, decreased cytotoxicity was also observed in the Δ*exsA*Δ*ndk-* and Δ*pscJ*Δ*ndk-*infected cells compared to Δ*ndk*-infected ones (Fig. 3), further indicating that T3SS might be involved in Ndk mediated *P. aeruginosa* virulence. Nevertheless, the cytotoxicity of Δ*exoS* and Δ*exoT* strains was decreased in comparison to that of PAO1 strain, while the cytotoxicity of Δ*exoS*Δ*ndk* and Δ*exoT*Δ*ndk* strains displayed no significant change compared to that of Δ*ndk* strain (Fig. 3), indicating that the increased cytotoxicity of Δ*ndk* was due to the combined cytotoxic effect of T3SS proteins.

**Figure 4:**
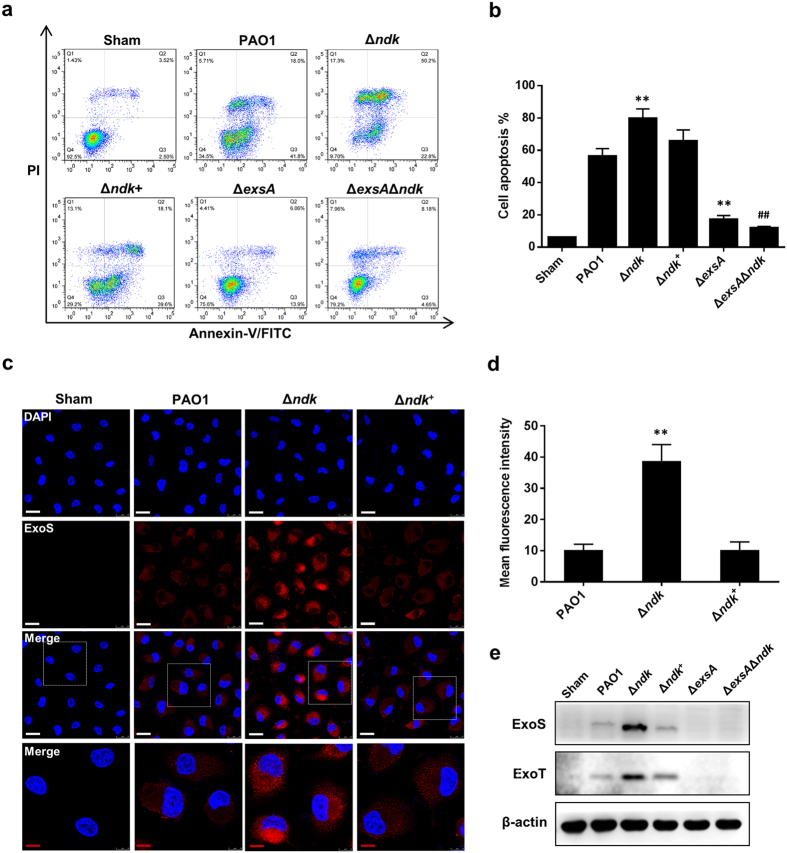
The *ndk* null-mutant promotes *P. aeruginosa* T3SS-mediated cell apoptosis. A549 cells were challenged with the indicated bacteria (MOI = 50) for 2 h. (**a**) Cell apoptosis was analyzed by flow cytometry using Annexin V-FITC-PI apoptosis detection kit. (**b**) Histogram represents the percentage of cell apoptosis from (**a**). (**c**) Examination of effector protein ExoS translocation by IF staining. White scale bar: 25 μm. Red scale bar: 10 μm. (**d**) Fluorescence intensity per cell from (**c**) was analyzed by IPP software (n_cell_ = 30). (**e**) Western blot analysis for intracellular translocation of ExoS. Representative images from triplicate experiments are shown. Data are expressed as mean ± SD from three independent experiments. *Indicates *P* < 0.05 compared to PAO1 strain. ^#^Indicates *P* < 0.05 compared to Δ*ndk* strain.

It has been shown that *P. aeruginosa* T3SS promotes cell apoptosis by injecting toxic effectors (such as ExoS) into the cytoplasm of host cells^29^; therefore, we detected cell apoptosis by flow cytometry and intracellularly translocated ExoS by immunofluorescence (IF) staining and western blotting in PAO1-, Δ*ndk-* or/and Δ*exsA*Δ*ndk*-infected cells. As shown in Fig. 4a,b, the Δ*ndk* strain promoted cell apoptosis compared to the PAO1 and Δ*ndk*^+^ strains, whereas apoptosis in the Δ*exsA*Δ*ndk-*infected cells decreased. Furthermore, the IF and western blot assays demonstrated that the intracellular translocation of ExoS was enhanced in Δ*ndk*-infected cells compared to PAO1- and Δ*ndk*^+^-infected cells (Fig. 4c–e), whereas the translocation of ExoS was decreased in the Δ*exsA*- and Δ*exsA*Δ*ndk*-infected cells (Fig. 4e).These results suggested that the null-mutation of *ndk* promotes cell apoptosis in a T3SS effector-dependent manner.

In addition, the T3SS translocation apparatus-mediated pore formation on the cell membrane is another determinant for cytotoxicity^5^,^7^. Hence, we further observed the cell membrane permeability after infection by observing calcein leakage via calcein AM-staining. This experiment confirmed that *ndk* deprivation promotes cytotoxicity by elevating cell membrane permeability (Supplementary Fig. 2).

### *Ndk* deficiency leads to up-regulated expression of *P. aeruginosa* T3SS

Given that T3SS consists of complex protein components^5^, the gene expression profiles of PAO1 and Δ*ndk* strains cultured in LB broth for 12 h were analyzed by mRNA transcriptome sequencing to obtain a comprehensive understanding of the role of Ndk for the T3SS. *Ndk* deprivation led to massive up-regulation of genes participating in the generation of T3SS components. These genes contain four gene clusters, PA1690-PA1697, PA1698-1709, PA1710-1712, and PA1714-1725, and effector protein genes *exoS, exoT,* and *exoY*, and chaperone *spcS* (Table 1).

**Figure 5:**
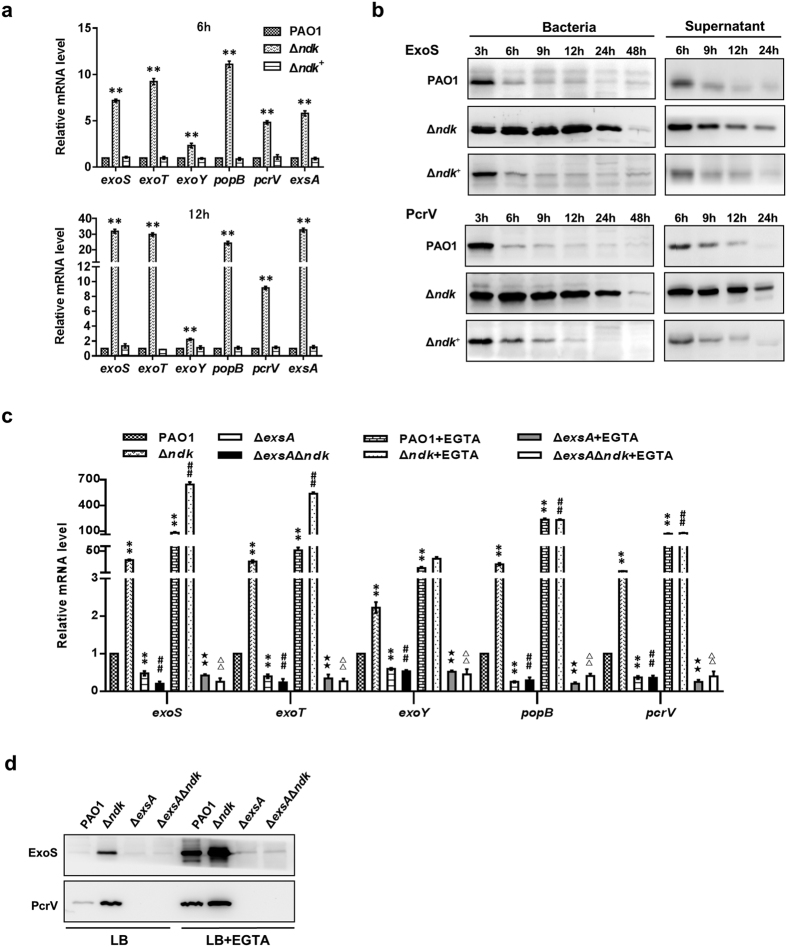
*Ndk* deficiency leads to up-regulated expression of *P. aeruginosa* T3SS. The indicated bacteria were inoculated into LB broth and a T3SS inducing condition (LB broth supplemented with 5 mM EGTA and 20 mM MgCl_2_) (initial OD_600_ of 0.01) and cultured at 37 °C for the indicated time. The bacteria and culture media were harvested for the subsequent analysis. (**a**) Real-time PCR analysis for T3SS-encoding gene expression. (**b**) Western blot analysis for the intracellular expression and secretion of T3SS proteins. (**c**) Real-time PCR analysis for T3SS gene expression with EGTA induction for 12 h. (**d**) Western blot analysis for intracellular T3SS protein expression with EGTA induction for 12 h. Representative images from triplicate experiments are shown (**b**,**d**). Data represent mean ± SD from three independent experiments (**a,c**). *Indicates *P* < 0.05 compared to PAO1 strain. ^#^Indicates *P* < 0.05 compared to Δ*ndk* strain. ★ Indicates *P* < 0.05 compared to PAO1 + EGTA group. Δ Indicates *P* < 0.05 compared to Δ*ndk* + EGTA group.

To further verify the results from mRNA transcriptome sequencing, the gene expression levels of T3SS of PAO1, Δ*ndk,* and Δ*ndk*^+^ strains cultured for 12 h in LB broth were analyzed by real-time PCR. In accordance with the results from transcriptome sequencing (Table 1), the genes encoding the translocator apparatus (PopB and PcrV), effectors (ExoS and ExoT) and the transcriptional regulator ExsA were all sharply increased in Δ*ndk* cells compared to PAO1 and Δ*ndk*^+^ cells at the early (6 h) and middle (12 h) bacterial growth phases (Fig. 5a) (the bacterial growth curve is presented in Supplementary Fig. 3). In addition, western blot analysis revealed that the intracellular and secretory protein levels of ExoS and PcrV of the Δ*ndk* strain were significantly higher than those in the PAO1 and Δ*ndk*^+^ strains (Fig. 5b). Taken together, these results indicated that Ndk might serve as a negative regulator controlling T3SS expression in *P. aeruginosa*.

### Ndk negatively regulates T3SS gene expression in an ExsA-dependent manner

ExsA is a master transcription factor for the activation of T3SS gene expression^30^. In accordance with the results that showed that a lack of *ndk* resulted in increased expression of *exsA* (Table 1; Fig. 5a,b), we investigated the role of ExsA in the Ndk-mediated regulation of T3SS. Hence, Δ*exsA* and Δ*exsA*Δ*ndk* double-knockout strains were generated and cultured for 12 h in LB broth, and gene and protein expression levels were analyzed by real-time PCR and western blot, respectively. As shown in Fig. 5c,d, the Δ*exsA*Δ*ndk* mutant displayed a distinct down-regulation of *exoS, exoT, exoY, popB, pcrV* and ExoS and PcrV compared to the Δ*ndk* mutant. Given that ExsA-dependent activation of T3SS was induced under a Ca^2+^-limiting growth condition^8^, the influence of ExsA on T3SS expression in Δ*ndk* cells was further investigated by adding a Ca^2+^ chelating agent (EGTA) to the bacterial culture medium. The addition of 5 mM EGTA markedly stimulated T3SS gene and protein expression levels in the PAO1 and Δ*ndk* strains rather than in the Δ*exsA* and Δ*exsA*Δ*ndk* strains (Fig. 5c,d), demonstrating that *ndk* negatively regulates T3SS expression in an ExsA-dependent manner.

### The deprivation of *ndk* inhibits the synthesis of exoproducts of *P. aeruginosa*

**Figure 6:**
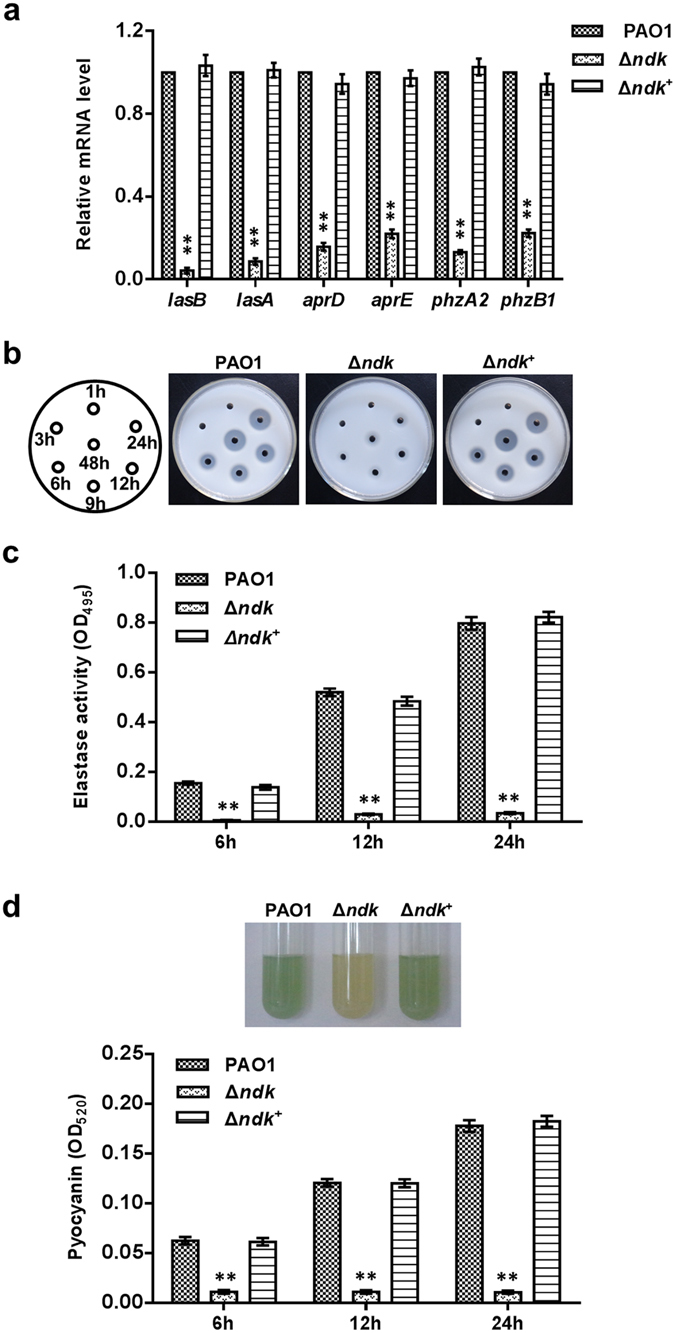
The deprivation of *ndk* inhibits the synthesis of exoproducts of *P. aeruginosa*. PAO1, Δ*ndk* and Δ*ndk*^+^ strains were inoculated into LB broth (initial OD_600_ of 0.01) and cultured at 37 °C for the indicated time. The bacteria and culture media were separately harvested for the subsequent analysis. (**a**) Real-time PCR analysis for exoproduct-related gene expression (12 h). (**b**) Skim milk plate assay for analysis of secreted proteases. (**c**) ECR assay for detecting elastase. (**d**) Analysis for pyocyanin production. Data represent mean ± SD from three independent experiments. *Indicates *P* < 0.05 compared to PAO1 strain.

In addition to T3SS, the exoproducts secreted by *P. aeruginosa* are also crucial virulence determinants for acute infection^3^. To investigate whether Ndk plays a role in regulating these products, the gene expression levels of those factors in PAO1, Δ*ndk* and Δ*ndk*^+^ strains cultured in LB broth for 12 h were evaluated. mRNA transcriptome sequencing revealed that the transcription levels of genes encoding exoproducts such as elastase (*lasA* and *lasB*), alkaline protease (*aprA* and *aprE*-*F*), phospholipase C (*plcB*) and phenazine (*phzA1*-*C1, phzE1, phzS* and *phzA2-C2*) were significantly impaired in Δ*ndk* cells compared to PAO1 cells (Table 1). In addition, real-time PCR analysis demonstrated a similar trend (Fig. 6a).

Subsequently, the secreted proteases from the bacterial culture media were estimated using a skim mild plate assay and an Elastin-Congo Red (ECR) assay. The activities of secreted proteases, including elastase, were significantly inhibited in Δ*ndk* cells compared to PAO1 and Δ*ndk*^+^ cells (Fig. 6b,c). Given that phenazine is an intermediate metabolic product for pyocyanin^31^, it was assumed that the gene changes in phenazine might affect pyocyanin synthesis. Therefore, we determined pyocyanin production in the bacterial culture medium. As shown in Fig. 6d, the Δ*ndk* strain produced barely detectable pyocyanin, whereas the Δ*ndk*^+^ strain produced pyocyanin at a level comparable to the PAO1 strain.

### *Ndk* negatively regulates the expression of the ExsA-mediated T3S regulon and positively modulates the expression of exoproducts through the QS system

**Figure 7:**
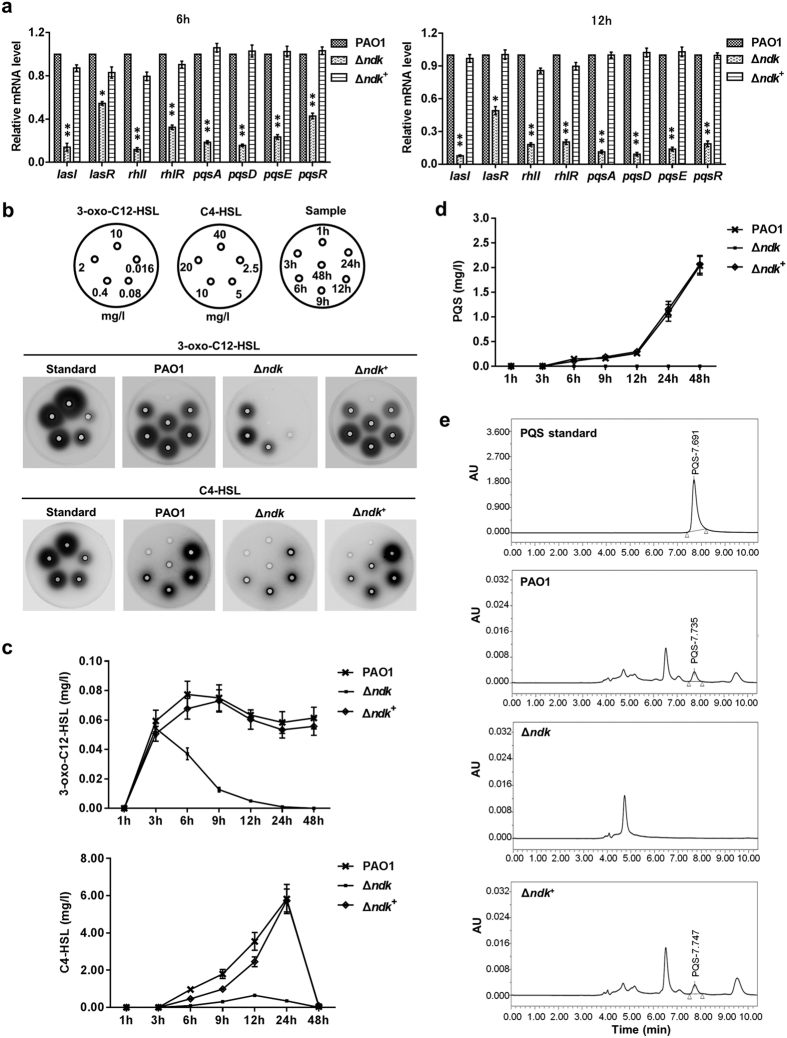
The deprivation of *ndk* inhibits quorum sensing hierarchy. PAO1, Δ*ndk* and Δ*ndk*^+^ strains were inoculated into LB broth (initial OD_600_ of 0.01) and cultured at 37 °C for the indicated time. The bacteria harvested at 6 h and 12 h were dissolved in TRIzol for gene expression analysis (**a**). The 3-oxo-C12-HSL and C4-HSL in the bacterial culture media were detected with a well-diffusion assay (**b**) and calculated by the measurement of gray value using Image J software (**c**). The PQS production in the bacterial culture medium was analyzed with HPLC (**d**) and the representative diagrams (12 h) were presented in (**e**). Data represent mean ± SD from three independent experiments. *Indicates *P* < 0.05 compared to PAO1 strain.

During acute pneumonia caused by *P. aeruginosa, ndk* gene expression was inhibited, while the expression levels of T3SS-encoding genes were activated in the BALFs of the infected mice (Fig. 1a). Furthermore, the expression levels of *lasI, rhlI,* and *pqsA,* which encode the QS synthetases, were also inhibited in that condition (Supplementary Fig. 4). These results suggest that QS might participate in the *ndk*-mediated regulation of T3SS upon infection. To address this issue, we set out to analyze the expression levels of QS genes in PAO1 and Δ*ndk* cells after culture in LB broth. Results from mRNA transcriptome sequencing showed that *lasI, rhlI, pqsABCDE, lasR, rhlR* and *pqsR* expression levels were all significantly inhibited in Δ*ndk* cells compared to PAO1 cells (Table 1). In addition, gene expression levels verified by real-time PCR also demonstrated decreased trends (Fig. 7a). Afterwards, signaling molecules in the bacterial culture media were further detected with a well-diffusion assay and a high-performance liquid chromatography (HPLC) assay. The null-mutation of *ndk* resulted in obvious inhibition of 3-oxo-C12-HSL and C4-HSL synthesis, whereas complementation of the *ndk* gene restored their production (Fig. 7b,c). The HPLC analysis revealed that PQS production in Δ*ndk* cells was apparently inhibited in comparison to PAO1 and Δ*ndk*^+^ cells (Fig. 7d,e).

**Figure 8:**
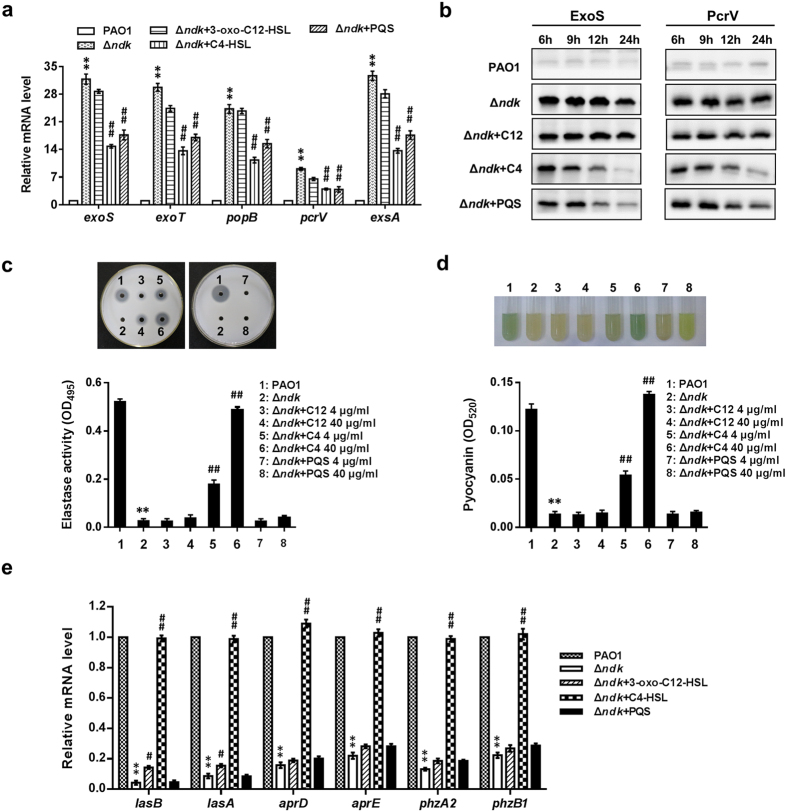
*Ndk* regulates the expression of the ExsA-mediated T3S regulon and exoproducts through the QS system. PAO1 and Δ*ndk* strains were inoculated into LB broth at an initial OD_600_ of 0.01. Exogenous 3-oxo-C12-HSL, C4-HSL or PQS (4 μg/ml or 40 μg/ml) was added to the culture medium of Δ*ndk* cells. The bacteria and culture supernatants were harvested for subsequent analysis at the indicated culture time. (**a**) Real-time PCR analysis for T3SS-encoding gene expression (signal molecules at 40 μg/ml, 12 h). (**b**) Western blot analysis for the intracellular expression of T3SS proteins (signal molecules at 40 μg/ml, 12 h). (**c**) Skim milk plate and ECR assays for secreted proteases (12 h). (**d**) Analysis for pyocyanin production (12 h). (**e**) Real-time PCR analysis for exoproduct-related gene expression (signal molecules at 40 μg/ml, 12 h). C12 indicate 3-oxo-C12-HSL. C4 indicates C4-HSL. Data represent mean ± SD from three independent experiments. *Indicates *P* < 0.05 compared to PAO1 strain. ^#^Indicates *P* < 0.05 compared to Δ*ndk* strain.

To examine whether decreased QS regulation might contribute to the increased expression of the T3S regulon in Δ*ndk* cells, exogenous 3-oxo-C12-HSL, C4-HSL or PQS at different concentrations were added to the culture medium of Δ*ndk* cells. The expression levels of *exoS, exoT, popB, pcrV, exsA* and ExoS and PcrV were reduced in Δ*ndk* cells when supplemented with C4-HSLs or PQS at 40 μg/ml (Fig. 8a,b). Supplementing with 40 μg/ml of 3-oxo-C12-HSL only slightly reduced the expression levels in Δ*ndk* cells; the protein levels did not significantly change (Fig. 8a,b). These results suggested that the *rhl* and *pqs* systems play more important roles than the *las* system in the regulation process of T3SS by Ndk in *P. aeruginosa*.

In addition to examining T3SS-encoding genes, we also analyzed the regulatory role of QS in the synthesis of exoproducts by adding exogenous 3-oxo-C12-HSL, C4-HSL or PQS to the culture medium of Δ*ndk* cells. Protease activity and pyocyanin production in Δ*ndk* cells were partially or completely recovered by the supplementation of C4-HSL at concentrations of 4 and 40 μg/ml, respectively (Fig. 8c,d). The exogenous addition of PQS and 3-oxo-C12HSL could not restore the elastase activity and pyocyanin production of Δ*ndk* cells even at a concentration of 40 μg/ml, even though an increase in pyoverdin production was observed in the Δ*ndk* +PQS (40 μg/ml) group (Fig. 8c,d). Moreover, gene expression analysis also revealed a similar trend (Fig. 8e). Taken together, our data suggest that *ndk* may negatively control bacterial virulence through the inhibition of T3SS transcription via the QS hierarchy in *P. aeruginosa*.

## Discussion

During infection, bacteria might alter gene expression to adapt to the host environment^32^. Adjusting gene expression enables bacterial survival, colonization and dissemination in the host, which might ultimately result in the enhancement of host pathogenesis^33^,^34^,^35^. Nevertheless, the underlying mechanism by which bacteria perceive host environmental stimuli and subsequently adjust remains poorly understood. The activation of bacterial stress response networks, such as the QS systems, two-component systems and regulatory genes may contribute to the bacterial adaptive response and bacterial virulence^13^,^33^,^34^,^36^. *soxR*, a gene encoding a superoxide response regulator, is highly induced during *P. aeruginosa* infection of mice with burn wounds, and increases in its expression promote bacteremia^33^. In addition, *suhB* (a metabolism-related gene)^34^ and PA3191^35^ were reported to be inducible genes of *P. aeruginosa* in the host pulmonary microenvironment, promoting mouse acute pneumonia through T3SS activation. In this study, we found that Ndk is a novel host-responsive regulator during acute infection and promotes *P. aeruginosa* virulence by suppressing its own expression, thereby triggering T3SS activation. Host cell contact is a way to modulate bacterial gene expression^8^. However, result demonstrated the host cell contact did not affect *P. aeruginosa ndk* gene expression (Supplementary Fig. 1). In the slime mould *Dictyostelium discoideum*, the sharply decreased transcript level of *ndk* gene is coinciding with the onset of the organism’s starvation-induced developmental cycle^37^,^38^, suggesting that host stress condition might play a role in modulating *ndk* gene expression. Upon lung infection, *P. aeruginosa* might encounter diverse pulmonary microenvironments including nutrition, iron, osmotic pressure and oxidative stress^39^,^40^. Whether these stimuli might act as the potential host factors that regulate *ndk* gene expression deserves further investigation.

T3SS consists of multiple protein components. It is reported that at least 42 genes are related to the T3S regulon, including five operons (36 genes) that participate in type III secretory machine generation and six additional genes encoding effectors and effector-specific chaperones^41^. In this study, we showed that Ndk exerts an extensive regulatory role on the T3SS (Table 1). ExsA, an AraC-like transcriptional regulator, plays a dominant role in the activation of T3S regulon transcription^9^. It has been shown that the five operons, including *exsD*-*pscL, exsCBA, pscG*-*popD, popN*-*pcrR*, and *pscN*-*pscU,* are all regulated by ExsA^41^. In addition, ExsA-binding sites have also been identified in the promoter regions of the effectors ExoS, ExoT, ExoY, ExoU, and the chaperone SpcS^1^. In this study, we demonstrated that *ndk* knockout results in the up-regulation of *exsA* and T3SS gene expression levels, whereas a null mutation of *exsA* from Δ*ndk* leads to suppressed expression of T3SS genes, indicating that Ndk regulates the transcription of T3SS genes in an ExsA-dependent manner (Fig. 5c,d). It has been shown that the human Ndk (NM23-H2), which shares highly homology with *E. coli* Ndk, is capable of binding to a nuclease-hypersensitive c-MYC promoter element and activating the c-MYC transcription^42^, indicating that bacterial Ndk might play a role in regulating gene transcription. Due to the fact that Ndk regulatesT3S regulon at the transcription level, it hints that Ndk might modulate T3SS in a promoter- or transcription factor-dependent manner.

T3SS activation is fine-tuned by a variety of factors, such as host cell contact, low Ca^2+^, metabolic signals, DNA damage, hyperosmotic stress and antibiotic exposure^8^. In addition, the QS hierarchy from *P. aeruginosa*^14^ and *V. harveyi (Vibrio harveyi*)^43^ also exhibits a negative effect on T3SS. In this study, we found that increased expression of *exoS, exoT, pcrV, popB*, ExoS and PcrV in Δ*ndk* cells was inhibited by C4-HSL or PQS rather than 3-oxo-C12HSL (Fig. 8a,b). Similar findings showed that the *exoS* gene was induced in the *rhlI, rhlR* or *pqsR* mutant, but not in the *lasI* mutant^44^,^45^. Bleves *et al*. demonstrated that the activity of a T3SS promoter fusion containing the secretion- and translocation-related operons increased in the *rhlI* mutant of *P. aeruginosa* but not in the *lasR* mutant^14^. In this study, we found that the C4-HSL mediated inhibition of T3SS genes in Δ*ndk* was due to *exsA* down-regulation (Fig. 8a). In *V. harveyi,* the QS regulator LuxR repressed T3SS gene expression through the inhibition of ExsA by binding to the P_*exsB*_ promoter region located upstream of the *exsBA* operon^43^, but the exact effect of RhlR/I-C4-HSL on ExsA in *P. aeruginosa* remains unclear.

In contrast to T3SS expression, the gene expression and synthesis of exoproducts in Δ*ndk* cells were globally reduced, including elastase (LasA and LasB) and pyocyanin (Fig. 6). This phenomenon was reversed by the addition of C4-HSL but not 3-oxo-C12HSL or PQS (Fig. 8c–e). It had been thought that all three QS systems contribute to the regulation of elastase and pyocyanin in *P. aeruginosa*^13^. Due to crosstalk between the three QS systems, that is, the inhibition of the *las* system might lead to suppressed *rhl* and *pqs* regulation^46^, the decreased elastase activity observed in the *lasI* mutant^47^ was considered to be due to the influence of the *rhl* system. Brint *et al*. also observed defective synthesis of elastase and pyocyanin in *rhlR* and *rhlI* mutants^48^, suggesting that Ndk mediates the inhibition of exoproducts through the *rhl* system. In addition, it was found that the deprivation of *ndk* gene led to deficient swarming, enhanced twitching motility (Supplementary Fig. 6) and decreased biofilm formation compared to PAO1 strain (Supplementary Fig. 7), which indicates that Ndk might act as a multifunctional protein to regulate bacterial virulence.

As an NTP-generating kinase, Ndk maintains NTP balance by catalyzing the reversible transfer of the γ-phosphate of NTP to NDP. In addition, Ndk functions as a histidine kinase to mediate γ-phosphate transfer from NTPs to itself and then to the substrate proteins, such as EnvZ and CheA^49^,^50^. The histidine 117 (His117) in the His-Gly-Ser-Asp (HGSD) motif, which is conserved among prokaryotic species, is vital for Ndk-mediated autophosphorylation and phosphotransfer activity^50^,^51^. To investigate whether Ndk-mediated virulence regulation is dependent on the Ndk phosphorylation activity, the kinase activity-deficient strain PA (H117Q) was constructed, and its related phenotypes were examined. Unexpectedly, the gene expression levels of T3SS, three QS synthetases and elastase did not show apparent changes in the PA (H117Q) mutant compared to the PAO1 strain (Supplementary Fig. 5a). The T3SS protein levels, AHL production, protease activity and pyocyanin production were also comparable between PAO1 and PA (H117Q) strains (Supplementary Fig. 5b–e), suggesting that Ndk-mediated virulence regulation may not rely on its kinase activity. Consistent with our finding, Neeld *et al*. showed that the *P. aeruginosa* Ndk H117Q mutant induces a similar level of cytotoxicity compared to Ndk when expressed inside host cells^23^.

In this study, it was shown that cytotoxicity were enhanced in Δ*ndk* cells, in which T3SS was activated while exoproduct synthesis was inhibited (Figs 2, 3, 4, 5, 6), suggesting that T3SS plays a dominant role in promoting host pathogenicity rather than the toxic exoproducts produced during acute infection. Upon pulmonary infection, T3SS promotes macrophage death and precludes macrophage-mediated phagocytosis to facilitate bacterial survival^5^, while the exoproducts disrupt epithelial barrier integrity and lyse fibrin to promote bacterial dissemination^3^. T3SS is activated at a low bacterial density, whereas it is inhibited at high bacterial density^43^, while the toxic exoproducts are secreted when bacteria reach a high density^47^. These different bacterial virulence regulatory patterns suggest that bacteria might activate T3SS for their survival in the host. Afterward, the bacterial cells might shut down T3SS and activate virulence product secretion for dissemination when they reach a high density. Because *ndk* expression is down-regulated upon acute infection, the opposite regulatory effects of Ndk on T3SS and exoproducts observed in this study imply that Ndk plays a critical role in adjusting bacterial host pathogenicity and adaptive responses.

**Figure 9:**
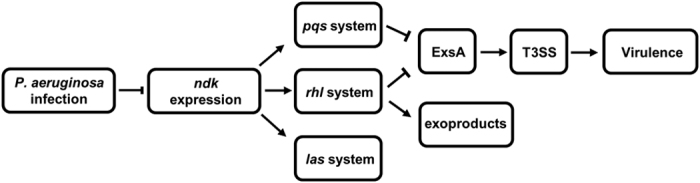
Schematic diagram of the proposed mechanism of Ndk-mediated virulence regulation. Upon *P. aeruginosa* infection, *ndk* expression was down-regulated in the pulmonary alveoli of mouse model of acute pneumonia. Knockout of *ndk* inhibited the *las, rhl* and *pqs* QS system. The inhibited *rhl* and *pqs* systems promoted T3SS gene and protein expression levels in an ExsA-dependent manner. The suppressed *rhl* system decreased exoproduct-related gene expression and product synthesis. In general, the inhibition of *ndk* promoted *P. aeruginosa* cytotoxicity and host pathogenicity through T3SS activation.

In conclusion, our study showed that *ndk* of *P. aeruginosa* is a novel host-responsive gene that is down-regulated during acute lung infection. *Ndk* down-regulation increased the expression of the T3S regulon in a *rhl*/*pqs* circuit-ExsA-dependent manner and suppressed exoproduct synthesis in a *rhl* system-dependent manner (Fig. 9). The inhibited expression of *ndk* enhanced cytotoxicity and host pathogenicity, implying that Ndk plays a crucial role in coordinating bacterial virulence and host adaptation during acute *P. aeruginosa* infection.

## Methods

### Bacterial strains, cells and animals

The details of the bacterial strains used in this study are listed in Supplementary Table 1. The *P. aeruginosa* strain PAO1 and its derivate mutants were grown in LB broth at 37 °C with shaking at 200 rpm. The AHL biosensor strains *E. coli* JM109/pSB1075 and JM109/pSB536, used for detection of QS signal molecules 3-oxo-C12-HSL and C4-HSL, respectively, were grown at 30 °C in LB broth. Antibiotics were used at the following concentrations: for *E. coli* JM109 (pSB1075), tetracycline 10 μg/ml, for *E. coli* JM109 (pSB536), ampicillin 50 μg/ml, for *P. aeruginosa*, carbenicillin 300 μg/ml, gentamicin 300 μg/ml, and kanamycin 300 μg/ml.

The A549 cells were purchased from ATCC and cultured in Dulbecco’s modified Eagle’s medium (DMEM, HyClone) supplemented with 10% (vol/vol) fetal bovine serum (FBS, Hyclone) in the presence of 5% CO_2_ at 37 °C.

BALB/c mice (6 to 8 weeks old, body weights of 20 to 25 g) were purchased from the Animal Center of Third Military Medical University (TMMU). All animal experiments were approved by the animal ethics committees of Xinqiao Hospital, TMMU and complied with the guidelines outlined in the National Institutes of Health Guide for the Care and Use of Laboratory Animals. All experiments performed in this study were approved by the Biosafety Committee of Xinqiao Hospital, TMMU.

### Plasmid and strain construction

DNA manipulation and plasmid construction were performed using standard molecular cloning technique. The lambda Red-based homologous recombination technique was employed to generate gene modification mutants of *P. aeruginosa* PAO1, using PCR fragment containing antibiotic cassette flanked by homology regions to the target genome locus^52^,^53^. The primers used in plasmid and strain construction were presented in Supplementary Table S2.

### Mouse acute pneumonia model

Bacteria cultured in LB broth for 12 h were collected and resuspended in phosphate-buffered saline (PBS) at a concentration of 1 × 10^10^ CFU/ml. Mice were anesthetized by intraperitoneal administration of pentobarbital sodium. Ten microliters of bacterial cells was then placed in each nostril of the mouse, resulting in inhalation of approximately 0.5 × 10^8^ bacteria per mouse. Control mice were treated with PBS in the same manner. After 7 days of infection, mice were sacrificed, and their lungs were removed for further analysis.

### Hematoxylin-eosin and immunohistochemistry staining

The mouse lungs were fixed with paraformaldehyde, embedded in paraffin and stained with H&E. Infiltrating leukocytes and macrophages were identified by IHC staining using antibodies against leukocyte common antigen CD45 (1:200, Santa Cruz) and macrophage antigen CD68 (1:200, Santa Cruz). The tissue sections were visualized using 3,3-diaminobenzidine (Zhongshan) and were observed under a light microscope (Olympus BX63).

### Cytotoxicity assay

A total of 1 × 10^4^ A549 cells were seeded in each well of a 96-well culture plate until they reached 80% confluence. The cells were infected with bacteria (MOI = 50) or bacterial culture media for 2 h. Cell viability was detected with a CCK-8 (Beyotime) according to the manufacturer’s instructions. In addition, the cell viability was determined by calcein-AM (Santa Cruz) staining^54^. A total of 1 × 10^5^ cells were cultured in cell culture dishes (15 mm) and infected with the indicated PAO1 strains (MOI = 50) for 2 h. The cells were loaded with 4 μm calcein-AM for 30 min in D-Hanks buffer at room temperature (RT), followed by 30 min incubation at RT. Image acquisition was performed with a Leica TCS SP5 confocal laser scanning microscope.

### Immunofluorescence staining

To determine the cellular location of the effector protein ExoS, A549 cells grown on coverslips were challenged with bacteria (MOI = 50) for 2 h at 37 °C, 5% CO_2_. Immunostaining was performed with the rat anti-ExoS antibody (generated in our laboratory), followed by hybridization with Alexa Fluor 594-labeled goat anti-rat IgG (1:200, Invitrogen). The cellular nuclei were stained with 4, 6-diamidino-2-phenylindole (DAPI) (1:1000, Beyotime). Images were acquired and analyzed using a Leica TCS SP5 laser confocal microscope.

### Determination of effector protein translocation

The translocation assay was based on an established protocol^55^. One day prior to bacterial infection, 1.5 × 10^6^ A549 cells were seeded in 100-mm-diameter tissue culture dishes. Then, the cells were challenged with bacteria (50 MOI) for 2 h at 37 °C, 5% CO_2._ After washing with PBS, 150 μl of 1% Triton X-100 was added to each plate, and cells were scraped from the dishes. The supernatants were harvested from the cell debris by centrifugation at 14,000 rpm for 15 min. The cellular translocation of the effector ExoS was detected by western blot.

### Flow cytometry analysis of cell apoptosis

A549 cells seeded in 6-well plates were infected with the indicated strains at an MOI of 50 for 2 h. Then, 1 × 10^6^ cells were harvested by trypsinization (0.25%) and stained with Annexin V-FITC and propidium iodide (PI) for 15 min at RT using a commercial apoptosis detection kit (BD Pharmingen). Stained cells were analyzed with a flow cytometer (MoFlo XDP, Beckman Coulter).

### Real-time PCR for gene expression

For gene expression analysis, the samples were dissolved in TRIzol (Takara Bio Inc.). Total RNA extraction and reverse transcription to cDNA were performed according to the manufacturer’s instructions. The genes were quantified by semi-quantitative real-time PCR. The gene expression levels were normalized to the housekeeping gene, *rplU*^56^. The primer sequences are presented in Supplementary Table S3.

### Transcriptome sequencing

For transcriptome sequencing, PAO1and Δ*ndk* cells were cultured in LB broth medium (initial OD_600_ of 0.01). After a 12-h culture, the bacterial cells were harvested and then dissolved in TRIzol (Takara Bio, Inc.). The series of experiments, including mRNA extraction, RNA fragmentation, cDNA synthesis and RNA-Seq library construction, were conducted by BGI Biotech Company, and libraries was sequenced using an Illumina HiSeq 2000 platform with the paired-end sequencing module.

### Western blot analysis for protein expression

The bacteria were inoculated into 300 ml of LB broth (initial OD_600_ of 0.01) and cultured at 37 °C for the indicated time. To induce T3SS-encoding gene expression, the bacteria were inoculated into LB broth supplemented with 5 mM EGTA and 20 mM MgCl_2_^57^. The bacterial proteins were quantitated using a BCA (bicinchonininc acid) protein assay kit. The secreted proteins in bacterial culture supernatants were harvested after precipitation with 30% trichloroacetic acid (TCA). Equal amounts of bacterial proteins or secreted proteins at the indicated times were resolved via sodium dodecyl sulfate-polyacrylamide gel electrophoresis (SDS-PAGE) and were subjected to western blot analysis using rat anti-ExoS, mouse anti-PcrV or/and mouse anti-Ndk polyclonal antibodies generated in our laboratory. The antibody signals were detected using an ECL plus kit (Thermo Fisher Scientific).

### Determination of extracellular virulence products

The *P. aeruginosa* PAO1 strains were inoculated into 300 ml of LB broth media (an initial OD_600_ of 0.01) and were cultured at 37 °C with shaking. The activities of extracellular proteases and elastase in the bacterial culture supernatants were determined by skim milk plate and Elastin-Congo Red (ECR) assays, respectively, as described previously^47^. Pyocyanin production in the supernatant was quantitated using the method described earlier^58^.

### Detection of QS signal molecules

The signal molecules in the supernatants of the strains were extracted with ethyl acetate (acidified with 0.5% formic acid) and dissolved in 50% methanol. For detecting the AHL signal molecules, a well-diffusion assay was performed and prepared as described previously^59^. The bioluminescent zones, an indicator for AHL concentration, were captured using an ImageQuant LAS 4000 mini (GE Healthcare Biosciences, USA). To detect PQS, the ethyl acetate-extracted signal molecules were subjected to HPLC analysis following a method described elsewhere with some modifications^60^. Briefly, the PQS was analyzed using a TSKgel ODS-100Z 5 μm HPLC column (4.6 × 250 mm, Tosoh Corporation) and was detected with a Waters 2998 Photodiode Array detector at 325 nm. The PQS was eluted at a flow rate of 1 ml/min in an isocratic elution model using a mobile phase made up of 86% methanol (1% glacial acetic acid acidified) and 14% water (1% glacial acetic acid acidified).

### Statistical analysis

Data were analyzed using the SPSS 16.0 software package (SPSS Inc.). The data are expressed as the means ± standard deviations (M ± SD). Statistical analyses were performed using Student’s *t*-test or analysis of variance (ANOVA) followed by Newman–Keuls test. Survival curves were estimated using the Kaplan-Meier method and were compared with the log-rank statistics. A significant difference was defined as a *P* value less than or equal to 0.05.

## Additional Information

**How to cite this article**: Yu, H. *et al*. Ndk, a novel host-responsive regulator, negatively regulates bacterial virulence through quorum sensing in *Pseudomonas aeruginosa. Sci. Rep.* **6**, 28684; doi: 10.1038/srep28684 (2016).

## Supplementary Material

Supplementary Information

## Figures and Tables

**Table 1:** Altered gene expression in Δ*ndk* compared to PAO1.

Gene locus	Name	Gene function	log_2_ ratio (Δ*ndk*/WT)	*P* vaule
T3SS				
PA1690	pscU	translocation protein in type III secretion	3.02	1.05E-20
PA1691	pscT	translocation protein in type III secretion	4.84	1.06E-23
PA1692	PA1692	translocation protein in type III secretion	4.37	1.11E-26
PA1693	pscR	type III secretion system protein	1.23	1.30E-18
PA1694	pscQ	type III secretion system protein	6.87	6.27E-106
PA1695	pscP	translocation protein in type III secretion	17.11	2.30E-204
PA1696	pscO	translocation protein in type III secretion	16.91	3.66E-77
PA1697	PA1697	type III secretion system ATPase	8.15	0
PA1698	popN	type III secretion outer membrane protein PopN	4.80	8.74E-160
PA1699	pcr1	hypothetical protein	17.30	5.92E-59
PA1700	pcr2	hypothetical protein	7.06	3.27E-41
PA1701	pcr3	hypothetical protein	16.28	2.10E-38
PA1702	pcr4	hypothetical protein	4.21	5.90E-19
PA1703	pcrD	type III secretory apparatus protein PcrD	2.02	9.36E-80
PA1704	pcrR	transcriptional regulator PcrR	1.91	3.68E-06
PA1705	pcrG	type III secretion regulator	5.24	1.90E-83
PA1706	pcrV	type III secretion protein PcrV	5.64	0
PA1707	pcrH	regulatory protein PcrH	6.31	1.94E-209
PA1708	popB	translocator protein PopB	5.50	0
PA1709	popD	translocator outer membrane protein PopD	5.99	0
PA1710	exsC	exoenzyme S synthesis protein C	4.05	0
PA1711	exsE	hypothetical protein	4.12	5.97E-235
PA1712	exsB	exoenzyme S synthesis protein B	4.42	2.33E-175
PA1713	exsA	transcriptional regulator ExsA	4.06	4.80E-157
PA1714	exsD	hypothetical protein	4.78	0
PA1715	pscB	type III export apparatus protein	5.84	2.10E-115
PA1716	pscC	type III secretion outer membrane protein PscC	3.51	4.36E-184
PA1717	pscD	type III export protein PscD	6.02	7.01E-76
PA1718	pscE	type III export protein PscE	5.21	3.45E-31
PA1719	pscF	type III export protein PscF	3.92	7.80E-50
PA1720	pscG	type III export protein PscG	4.01	3.03E-46
PA1721	pscH	type III export protein PscH	3.95	2.41E-33
PA1722	pscI	type III export protein PscI	4.28	3.92E-39
PA1723	pscJ	type III export protein PscJ	3.06	2.39E-47
PA1724	pscK	type III export protein PscK	3.94	9.94E-12
PA1725	pscL	type III secretion system protein	2.49	2.20E-16
PA3841	exoS	exoenzyme S	3.69	0
PA0044	exoT	exoenzyme T	4.54	0
PA2191	exoY	adenylate cyclase	2.97	2.05E-46
PA3842	spcS	chaperone	3.69	1.27E-68
QS signal molecule synthetase and regulator				
PA1432	lasI	autoinducer synthesis protein LasI	−3.12	0
PA3476	rhlI	autoinducer synthesis protein RhlI	−2.09	0
PA0996	pqsA	coenzyme A ligase	−5.81	0
PA0997	pqsB	alkyl quinolones biosynthesis protein	−6.96	0
PA0998	pqsC	alkyl quinolones biosynthesis protein	−5.91	4.61E-260
PA0999	pqsD	3-oxoacyl-synthase III	−4.22	0
PA1000	pqsE	Quinolone signal response protein	−4.12	8.01E-254
PA2587	pqsH	FAD-dependent monooxygenase	−4.45	0
PA1430	lasR	transcriptional regulator LasR	−1.03	1.42E-240
PA3477	rhlR	transcriptional regulator RhlR	−3.39	0
PA1003	mvfR	transcriptional regulator MvfR (PqsR)	−1.31	6.81E-64
PA1898	qscR	quorum-sensing control repressor	3.32	7.68E-65
Virulence associated exoproducts				
PA3724	lasB	elastase LasB	−6.76	0
PA1871	lasA	LasA protease	−7.03	0
PA1246	aprD	alkaline protease secretion protein AprD	−2.92	2.19E-116
PA1247	aprE	alkaline protease secretion protein AprE	−2.95	5.20E-102
PA1248	aprF	alkaline protease secretion outer membrane protein AprF	−2.50	3.21E-55
PA1249	aprA	alkaline metalloproteinase	−1.25	0
PA0026	plcB	phospholipase C	−2.43	1.02E-219
PA4210	phzA1	phenazine biosynthesis protein	−0.98	0.051979
PA4211	phzB1	phenazine biosynthesis protein	−2.21	1.12E-53
PA4212	phzC1	phenazine biosynthesis protein PhzC	−1.81	3.68E-06
PA4214	phzE1	phenazine biosynthesis protein PhzE;	−2.42	4.68E-08
PA4217	phzS	flavin-containing monooxygenase	−2.05	8.77E-114
PA1899	phzA2	phenazine biosynthesis protein	−3.04	1.79E-22
PA1900	phzB2	phenazine biosynthesis protein	−4.89	0
PA1901	phzC2	phenazine biosynthesis protein PhzC	−4.26	1.81E-58
PA1130	rhlC	rhamnosyltransferase	−3.18	0
PA3478	rhlB	rhamnosyltransferase subunit B	−7.00	0
PA3479	rhlA	rhamnosyltransferase subunit A	−6.69	0
PA2570	lecA	PA-I galactophilic lectin	−5.82	0
