# Supplementary material for: Ndk, a novel host-responsive regulator, negatively regulates bacterial virulence through quorum sensing in Pseudomonas aeruginosa
Source: Sci Rep. 2016 Jun 27;6:28684. doi: 10.1038/srep28684 (PMC4921839; doi:10.1038/srep28684)
Supplement: Supplementary Information [file srep28684-s1.pdf]

Supplementary information

Ndk, a novel host-responsive regulator, negatively regulates bacterial virulence through quorum sensing in *Pseudomonas aeruginosa*

Hua Yu, Junzhi Xiong, Rong Zhang, Xiaomei Hu, Jing Qiu, Di Zhang, Xiaohui Xu, Rong Xin, Xiaomei He, Wei Xie, Halei Sheng, Qian Chen, Le Zhang, Xiancai Rao & Kebin Zhang

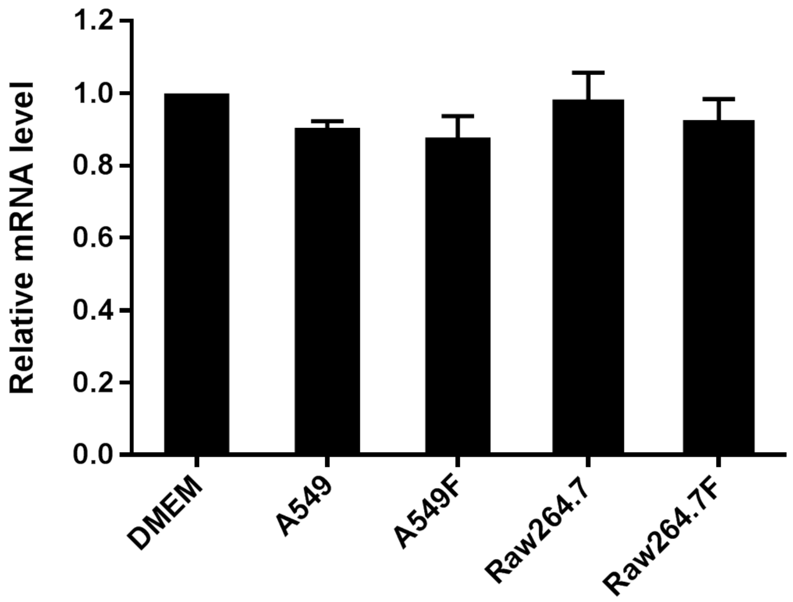

Supplementary Fig. 1 Real-time PCR analysis for *ndk* expression upon *P. aeruginosa* infection. A549 cells and RAW264.7 cells cultured in 12-well culture plate were fixed by 4% paraformaldehyde for 10 min at RT. The fixed and non-fixed cells were infected with PAO1 strain in DMEM (MOI=50) for 2 h. The PAO1 strain cultured in DMEM was used as control. A549F and RAW264.7F indicate fixed A549 cells and fixed RAW264.7 cells, respectively. Data are expressed as mean ± SD from triplicate experiments.

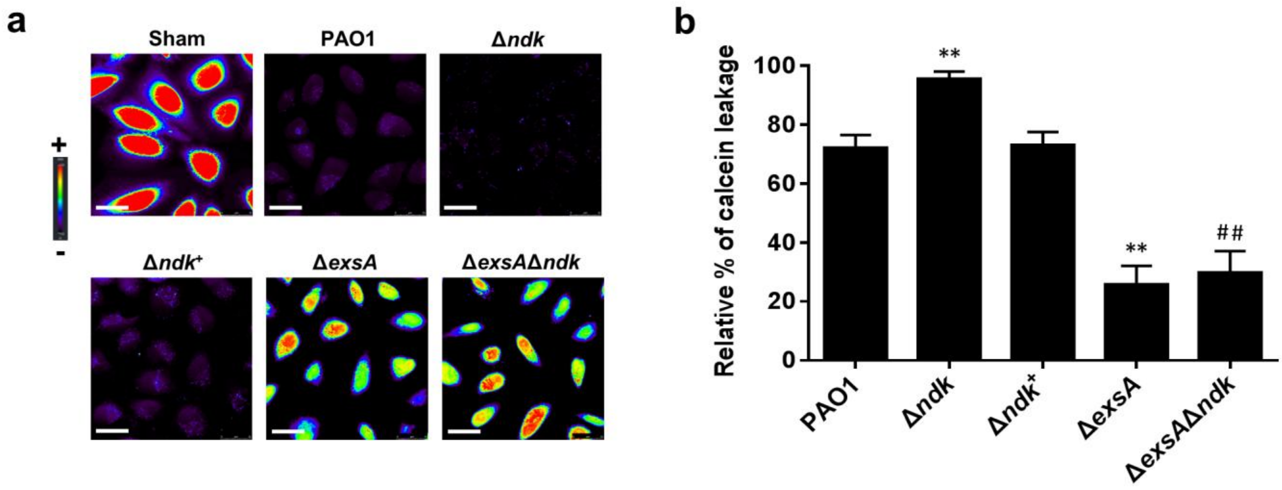

Supplementary Fig. 2 Calcein leakage assay. A549 cells grown in cell culture dishes (15 mm) were loaded with 4 μm calcein-AM for 30 min in D-Hanks buffer at RT. After three washes, cells were challenged with bacteria (MOI=50) for 1h at 37℃. Images were acquired using laser confocal microscope. (a) Representative fluorescence micrographs. Scale bar, 25 μm. (b) Relative percentage of calcein leakage was calculated after normalization to sham infected cells (n_{cell} =30). Data are expressed as mean ± SD from triplicate experiments. * indicates $P < 0.05$ compared to PAO1 strain. # indicates $P < 0.05$ compared to Δ*ndk* strain.

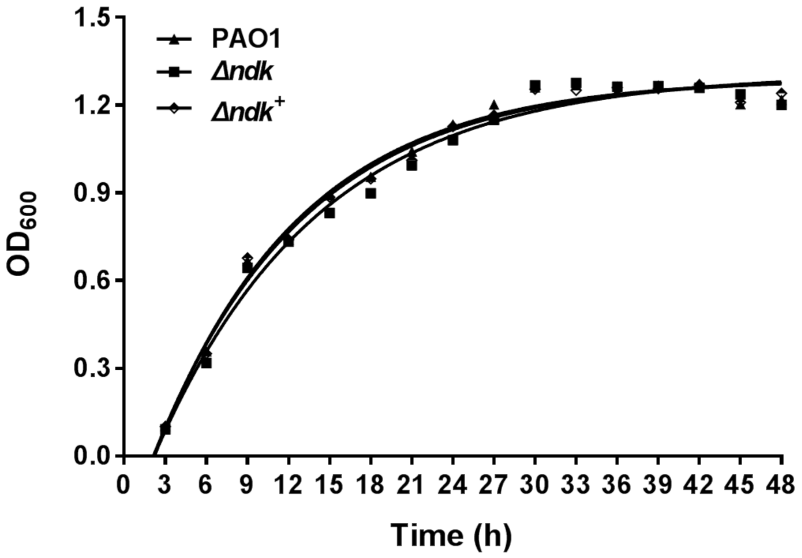

Supplementary Fig. 3 Assays for bacterial growth. PAO1, Δ*ndk* and Δ*ndk*$^+$ strains were inoculated into LB broth at an initial OD_{600} of 0.01 and cultured at 37℃ with shaking for 48 h. Three milliliter of bacterial culture was harvested and centrifuged after an interval time. The cell pellets were resuspended in the same volume of PBS and bacterial growth was monitored by measuring the OD_{600}. Data represent mean ± SD from three independent experiments.

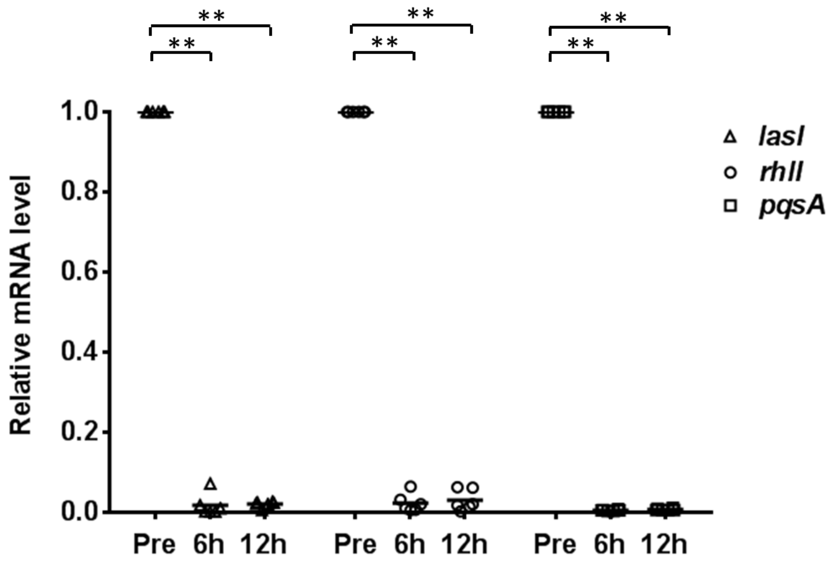

Supplementary Fig. 4 Transcription of QS synthetase genes was inhibited in a mouse model of acute pneumonia. Mice were intranasally challenged with 2×10^8 CFU of PAO1. At 6 and 12 h post-infection, the BALFs were collected from the infected mice and the expression levels of *lasI*, *rhlI* and *pqsA* in BALFs were evaluated by real-time PCR. The 50S ribosomal protein-coding gene *rplU* was used as an internal control. Black bars represent medians for the group of mice. In each time point, six mice were used. * represents $P < 0.05$ compared to bacteria *in vitro* (Pre).

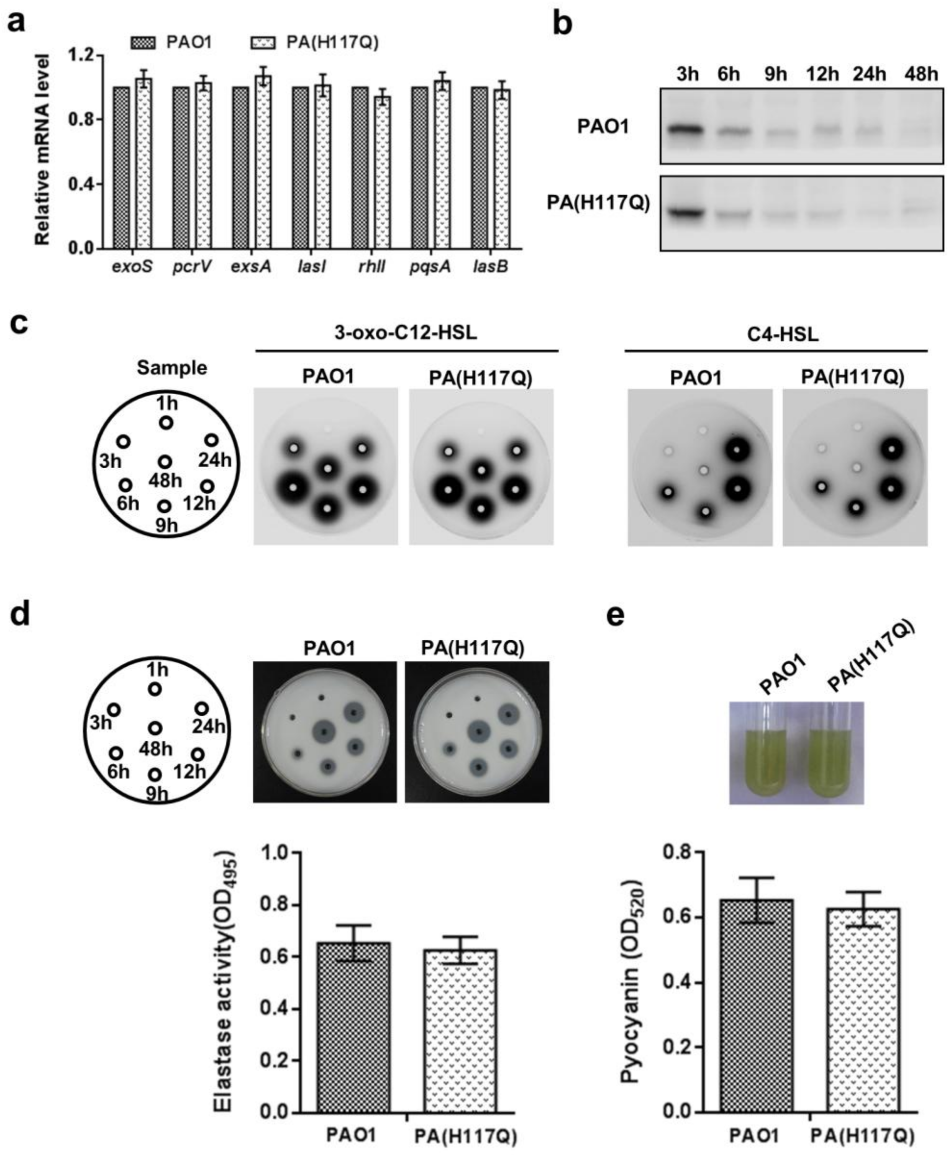

Supplementary Fig. 5 Ndk-mediated virulence regulation is not dependent on the Ndk phosphorylation activity. PAO1 and PA (H11Q) strains were inoculated into LB broth at an initial OD_{600} of 0.01 and cultured at 37℃ with shaking for the indicated time. (a) Real-time PCR analysis for gene expression (12 h). (b) Western blot analysis for the intracellular expression of T3SS effector protein ExoS. (c) Well-diffusion assay for the detection of 3-oxo-C12-HSL and C4-HSL in the bacterial culture media. (d) Skim milk plate for detecting secreted proteases and ECR assays for detecting elastase activity (12 h). (e) Assay for pyocyanin production (12 h). Data represent mean ± SD from three independent experiments. * indicates $P < 0.05$ compared to PAO1 strain.

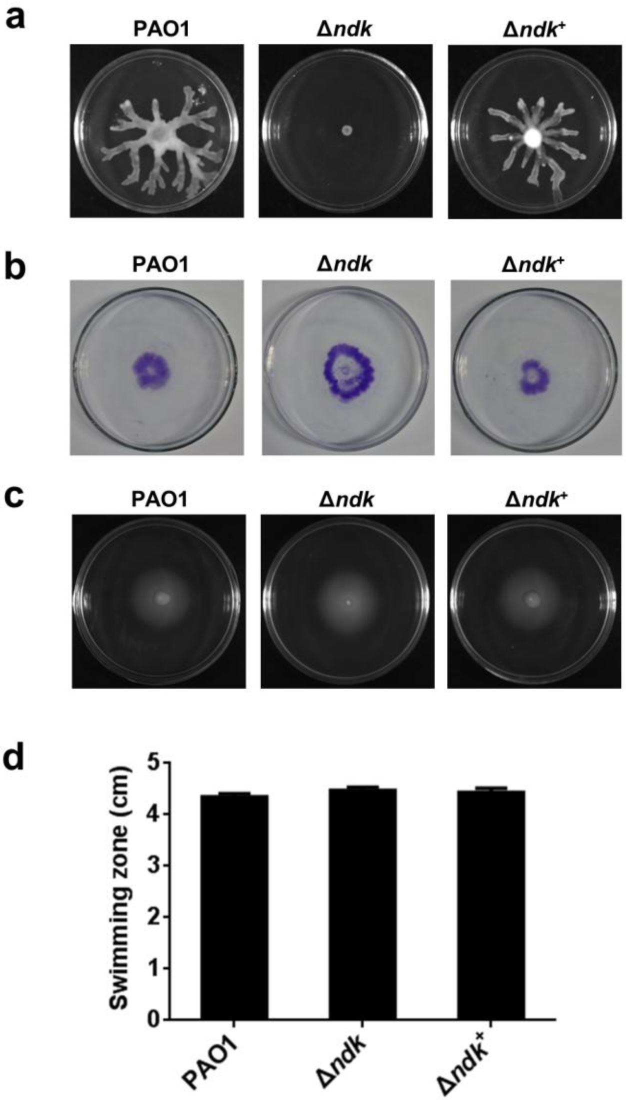

Supplementary Fig. 6 Motility Assays. For swarming or swimming motility assay, 1 μl of the indicated bacteria were inoculated on the central of the corresponding motility plates, and the plates were incubated at 37℃ for 24 h. (a) Swarming motility. Plates contain 0.8% Difco nutrient broth (wt/vol), 0.5% Difco bacto-agar (wt/vol) and 0.5% glucose (wt/vol). (b) Twitching motility assay. A single colony was inoculated with a toothpick to the bottom of the plastic plate containing 1% tryptone (wt/vol), 0.5% yeast extract (wt/vol), 1% NaCl (wt/vol) and 1% agar (wt/vol), and the plates were incubated at 37℃ for 48 h. The twitching motility on the surface of the plate was visualized by 1% crystal violet staining. (c) Swimming motility. Plates contain 1% tryptone (wt/vol), 0.5% NaCl (wt/vol) and 0.3% bacto-agar (wt/vol). (d) Swimming zone. Swimming zone was calculated by the measurement of swimming halo diameter. Data represent mean ± SD from three independent experiments.

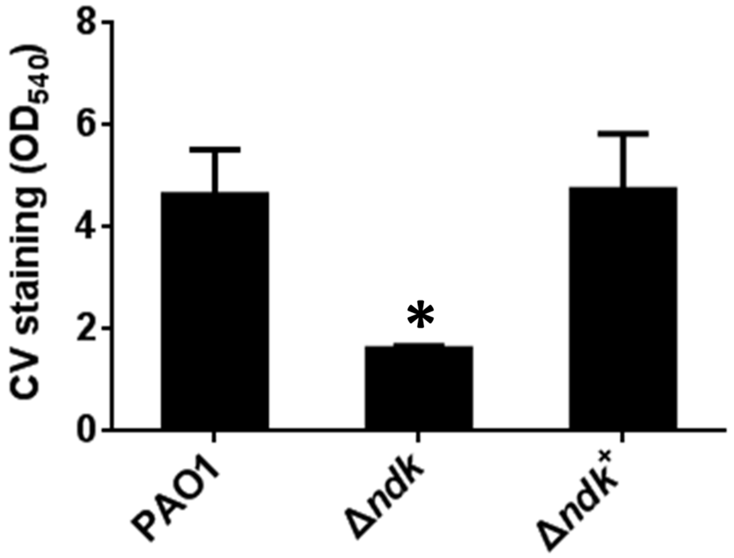

Supplementary Fig. 7 Biofilm quantitation. The indicated bacteria which were adjusted to OD_{600} of 0.05 with LB broth were filtrated on a polycarbonate membrane (Millipore, USA).The membranes were plated on MHB plates and incubated at 37℃ for 48 h without shaking. The biofilm formation on polycarbonate membrane was quantified by 1% crystal violet staining. Data represent mean ± SD from three independent experiments. * indicates $P < 0.05$ compared to PAO1 strain.

Supplementary Table S1. Strains and plasmids used in this study

Strain	Description	Source (Reference)
E. coli		
JM109/pSB1075	*lasR* + *lasI*::luxCDABE, Tc^r	[1]
JM109/pSB536	*rhlR* + *rhlI*::luxCDABE, Ap^r	[1]
BL21/pet28a	Kan^r	Invitrogen
JM109/pQE31	Ap^r	Qiagen
BL21/pet28a-exoS	Kan^r	This study
JM109/pQE31-pcrV	Ap^r	This study
P. aeruginosa		
PAO1	*P. aeruginosa* PAO1 strain contains pUCP-red plasmid, secretes ExoS, ExoT and ExoY effectors	This study
Δ*ndk*	PAO1 *ndk* was disrupted by replacement of Kan^r cassette in genome; Kan^r	This study
Δ*ndk*$^+$	*ndk*-Gm^r gene cassette was complemented to Δ*ndk* genome, Kan^r, Gm^r	This study
Δ*exsA*	PAO1 Δ*exsA* with *exsA* deletion, Tc^r	This study
Δ*pscJ*	PAO1 Δ*pscJ* with *pscJ* deletion, Tc^r	This study
Δ*exoS*	PAO1 Δ*exoS* with *exoS* deletion, Gm^r	This study
Δ*exoT*	PAO1 Δ*exoT* with *exoT* deletion, Gm^r	This study
Δ*exsA*Δ*ndk*	PAO1 Δ*ndk* with *exsA* deletion, Kan^r, Tc^r	This study
Δ*pscJ*Δ*ndk*	PAO1 Δ*ndk* with *pscJ* deletion, Kan^r, Tc^r	This study
Δ*exoS*Δ*ndk*	PAO1 Δ*ndk* with *exoS* deletion, Kan^r, Gm^r	This study
Δ*exoT*Δ*ndk*	PAO1 Δ*ndk* with *exoT* deletion, Kan^r, Gm^r	This study
PA(H117Q)	The 117 histidine residue of PAO1Ndk was replaced by glutamine, Kan^r	This study
**Plasmids**		
pUCP	*E. coli*-*P. aeruginosa* shuttle vector, Cb^r	[2]
pKD46	Lambda Red protein (Exo, Bet, Gam) expression vector, arabinose-inducible promoter, Amp^r	[3]
pUCP-red	*E. coli*-*P. aeruginosa* shuttle vector expressing lambda Red proteins, arabinose-inducible promoter, Cb^r	This study
pet28a-exoS	Kan^r	This study
pQE31-pcrV	Ap^r	This study

References

1. Winson MK, Swift S, Fish L, Throup JP, Jorgensen F, Chhabra SR, Bycroft BW, Williams P, Stewart GS: **Construction and analysis of luxCDABE-based plasmid sensors for investigating N-acyl homoserine lactone-mediated quorum sensing**. *FEMS Microbiol Lett* 1998, **163**(2):185-192.
2. Cronin CN, McIntire WS: **pUCP-Nco and pUCP-Nde: Escherichia-Pseudomonas shuttle vectors for recombinant protein expression in Pseudomonas**. *Anal Biochem* 1999, **272**(1):112-115.
3. Datsenko KA, Wanner BL: **One-step inactivation of chromosomal genes in Escherichia coli K-12 using PCR products**. *Proc Natl Acad Sci U S A* 2000, **97**(12):6640-6645.

Supplementary Table S2. Primers used for plasmid and strain construction

Plasimd/strain	Backgroud strain	Primer	Primer sequence
pUCP-red		red-F	5'ggcccatatgattatgacaacttgacggctac3'
		red-R	5'ggcctctagattcttcgtctgtttctactggt3'
pQE31-exoS		exoS-F	5'tataggatccgcatattcaatcgcttcagc3'
		exoS-R	5'atgcaagctttcaggccagatcaaggc3'
pET28a-pcrV		pcrV-F	5'tataggatccatggaagtcagaaaccttaatg3'
		pcrV-R	5'tataaagcttttagtggtggtggtggtggtggatcgcgctgagaatgtc3'
Δ*ndk*	PAO1/pUCP-red	Forward arm-F	5'catccagctcgacgaaagc3'
		Forward arm-R	5'gcgtcagaccccgtagaaaggttgtctagcatgggcg3'
		Kan^r-F	5'cgcccatgctagacaacctttctacggggtctgacgc3'
		Kan^r-R	5'tggtttcaccctcacccaccgcggaacccctatttgt3'
		Back arm-F	5'acaaataggggttccgcggtgggtgagggtgaaacca3'
		Back arm-R	5'gcagaaactgcaatccagg3'
Δ*ndk*$^+$	Δ*ndk*	Forward arm-F	5'ccgacaactggattgaccc3'
		Forward arm-R	5'cggcaaatgcgtagggccgtggcgatctgtctcatg3'
		ndk-F	5'catgagacagatcgccacggccctacgcatttgccg3'
		ndk-R	5'gtgccttcatccgtttcctcagcgaatgcgctcgc3'
		Gm^r-F	5'gcgagcgcattcgctgaggaaacggatgaaggcac3'
		Gmr-R	5'gagtgcgtggatcgtcgcttaggtggcggtacttggg3'
		Back arm-F	5'ccaagtaccgccacctaagcgacgatccacgcactc3'
		Back arm-R	5'acatcgcatttttcgcgc3'
Δ*exsA*	PAO1/pUCP-red	Forward arm-F	5'cccggaagaaagatctggc3'
		Forward arm-R	5'aagctgtcaaacatgagaaagaaccccaacacttcccgt3'
		Tet^r-F	5'acgggaagtgttggggttctttctcatgtttgacagctt3'
		Tet^r-R	5'accgggctttcaaaaaacgtcaggtcgaggtggcccg3'
		Back arm-F	5'cgggccacctcgacctgacgttttttgaaagcccggt3'
		Back arm-R	5'aggagaatctgccgcacct3'
Δ*pscJ*	PAO1/pUCP-red	Forward arm-F	5'acagccacaaactcgacctg3'
		Forward arm-R	5'atgataagctgtcaaacatgagaatgggtcttcatcagggtttc3'
		Tet^r-F	5'gaaaccctgatgaagacccattctcatgtttgacagcttatcat3'
		Tet^r-R	5'caactggtaggccgtcaatgttccattcaggtcgaggtg3'
		Back arm-F	5'cacctcgacctgaatggaacattgacggcctaccagttg3'
		Back arm-R	5'atgagcaggtcgagctgct3'
Δ*exoS*	PAO1/pUCP-red	Forward arm-F	5'tctgctcgttggccgtagc3'
		Forward arm-R	5'ggcaaccttgggcagcagcgatggttgccttctcctgat3'
		Gm^r-F	5'atcaggagaaggcaaccatcgctgctgcccaaggttgcc3'
		Gmr-R	5'gtctttcttttacgaccggttaggtggcggtacttgggt3'
		Back arm-F	5'acccaagtaccgccacctaaccggtcgtaaaagaaagac3'
		Back arm-R	5'agggcgccgagctgtactg3'
Δ*exoT*	PAO1/pUCP-red	Forward arm-F	5'gtgcacaggctcaacaacaga3'
		Forward arm-R	5'ggcaaccttgggcagcagcgatgattgacgtctcctgatg3'
		Gm^r-F	5'catcaggagacgtcaatcatcgctgctgcccaaggttgcc3'

		Gmr-R	5'cttgggagtgtccgtctctttaggtggcggtacttgggt3’
		Back arm-F	5'gacccaagtaccgccacctaaagagacggacactcccaag3’
		Back arm-R	5'aaggcgaggggcgaaatgg3’
Δ*exsA*Δ*ndk*	Δ*ndk*	Forward arm-F	5'cccggaagaaagatctggc3'
		Forward arm-R	5'aagctgtcaaacatgagaaagaaccccaacacttcccgt3'
		Tet^r-F	5'acgggaagtgttggggttctttctcatgtttgacagctt3'
		Tet^r-R	5'accgggctttcaaaaaacgtcaggtcgaggtggcccg3'
		Back arm-F	5'cgggccacctcgacctgacgttttttgaaagcccggt3'
		Back arm-R	5'aggagaatctgccgcacct3'
Δ*pscJ*Δ*ndk*	Δ*ndk*	Forward arm-F	5'acagccacaaactcgacctg3’
		Forward arm-R	5'atgataagctgtcaaacatgagaatgggtcttcatcagggtttc3’
		Tet^r-F	5'gaaaccctgatgaagacccattctcatgtttgacagcttatcat3’
		Tet^r-R	5'caactggtaggccgtcaatgttccattcaggtcgaggtg3’
		Back arm-F	5'cacctcgacctgaatggaacattgacggcctaccagttg3’
		Back arm-R	5'atgagcaggtcgagctgct3’
Δ*exoS*Δ*ndk*	Δ*ndk*	Forward arm-F	5'tctgctcgttggccgtagc3’
		Forward arm-R	5'ggcaaccttgggcagcagcgatggttgccttctcctgat3’
		Gm^r-F	5'atcaggagaaggcaaccatcgctgctgcccaaggttgcc3’
		Gmr-R	5'gtctttcttttacgaccggttaggtggcggtacttgggt3’
		Back arm-F	5'acccaagtaccgccacctaaccggtcgtaaaagaaagac3’
		Back arm-R	5'agggcgccgagctgtactg3’
Δ*exoT*Δ*ndk*	Δ*ndk*	Forward arm-F	5'gtgcacaggctcaacaacaga3’
		Forward arm-R	5'ggcaaccttgggcagcagcgatgattgacgtctcctgatg3’
		Gm^r-F	5'catcaggagacgtcaatcatcgctgctgcccaaggttgcc3’
		Gmr-R	5'cttgggagtgtccgtctctttaggtggcggtacttgggt3’
		Back arm-F	5'gacccaagtaccgccacctaaagagacggacactcccaag3’
		Back arm-R	5'aaggcgaggggcgaaatgg3’
PA(H117Q)	PAO1/pUCP-red	Forward arm-F-1	5’atggcactgcaacgcacc3
		Forward arm-R-1	5’gaagcttcggaatcggaaccttggacggcgttctcgtcg3’
		Forward arm-F-2	5’cgacgagaacgccgtccaaggttccgattccgaagcttc3’
		Forward arm-R-2	5’gcgtcagaccccgtagaatcagcgaatgcgctcgc3’
		Kanr-F	5’gcgagcgcattcgctgattctacggggtctgacgc3’
		Kanr-R	5’tggtttcaccctcacccaccgcggaacccctatttgt3’
		Back arm-F	5’acaaataggggttccgcggtgggtgagggtgaaacca3’
		Back arm-R	5’gcagaaactgcaatccagg3’

Supplementary Table S3. Primers used in real-time PCR

Primer	Sequence
rplU-RT-F	5'-cgcagtgattgttaccggtg-3'
rplU-RT-R	5'-ggtaaccttcgcaccttcga-3'
ndk-RT-F	5'-accctgtccatcatcaagcc-3'
ndk-RT-F	5'-gaacggacgctctttgtgc-3'
exoS-RT-F	5'-gacgcaagcccggaact-3'
exoS-RT-R	5'-caggctgtctgcccaggtac-3'
exoT-RT-F	5'-tcgaggcttcccgtaccca-3'
exoT-RT-R	5'-cagggcgaccttgtccatt-3'
exoY-RT-F	5'-tggtggacgccctcaatg-3'
exoY-RT-R	5'-gactcttctccgacccgatg-3'
pcrV-RT-F	5'-gatcgacgctggcggtat-3'
pcrV-RT-R	5'-tcatcgctgaggcccttg-3'
popB-RT-F	5'-gcgcttcgacgctgttgt-3'
popB-RT-R	5'-ttcttccgactccctgatcttct-3'
exsA-RT-F	5'-gatgctcgcctgcctgaa-3'
exsA-RT-R	5'-cgaactcgcgggagaagt-3'
lasI-RT-F	5'-ccgtttcgccatcaact-3'
lasI-RT-R	5'-tgccgatcttcaggtgc-3'
lasR-RT-F	5'-tcctgttcggcctgttgc-3'
lasR-RT-R	5'-gctgctttcgcgtctggt-3'
rhlI-RT-F	5'-tacctgtgcagcgaaaccc-3'
rhlI-RT-R	5'-gccgttgcgaacgaaatag-3'
rhlR-RT-F	5'-cgctcctcggaaatggtg-3'
rhlR-RT-R	5'-gctggagatgttctgctggtc-3'
pqsA-RT-F	5'-tgagcggtcctttggc-3'
pqsA-RT-R	5'-ggaacccgaggtgtattgc-3'
pqsD-RT-F	5'-ctgggcaacatggcttcg-3'
pqsD-RT-R	5'-cctcctcaggtttgcggta-3'
pqsE-RT-F	5'-ccacagcgacgatcacg-3'
pqsE-RT-R	5'-gactccaggtaagcctccat-3'
pqsR-RT-F	5'-tctgcgatacggtgagc-3'
pqsR-RT-R	5'-tttccgcgttgtcctg-3'
lasB-RT-F	5'-atgttctatccgctggtgtcg-3'
lasB-RT-R	5'-gctgcccttcttgatgtcgt-3'
lasA-RT-F	5'-atgacgacctgttcctctacgg-3'
lasA-RT-R	5'-tcagggtcagcaacactttcg-3'
aprD-RT-F	5'-caaggctcggttccagcgttac-3'
aprD-RT-R	5'-cgccatcagggtcagcatcaa-3'
aprE-RT-F	5'-ggcggaaacccaggtcaat-3'
aprE-RT-R	5'-tccaggctgtcgctgttgg-3'
phzA2-RT-F	5'-actgggagtggcacaacg-3'
phzA2-RT-R	5'-gcaatttctgcatcgggtt-3'
phzB1-RT-F	5'-acggctgtggcggttta-3'
phzB1-RT-R	5'-ccgtgaccgtcgcatt-3'
